# Bioindicators of Plastic Pollution: Insights into the Relationship between Environmental Plastic Abundance and Ingestion by Green Turtles ()

**DOI:** 10.1021/acs.est.5c01171

**Published:** 2025-07-08

**Authors:** Robson G Santos, Adriano Carvalho Vasconcelos, Priscilla Monteiro de Oliveira, João Paulo Felix Augusto de Almeida, Ingredy Silva, Bruno Stefanis S.P. de Oliveira, Matthew S. Savoca, Guilherme Ramos Demetrio

**Affiliations:** a ECOA LAB, Instituto de Ciências Biológicas e da Saúde, 28112 Universidade Federal de Alagoas, Maceió 57072-970, Brazil; b Instituto Biota de Conservação, Maceió 57038-770, Brazil; c Hopkins Marine Station, Stanford University, Pacific Grove, California 93950, United States; d California Marine Sanctuary Foundation, Monterey, California 95061, United States; e Plant Ecology Lab (LEVE), Universidade Federal de Alagoas, Campus Arapiraca, Unidade Educacional Penedo, Penedo 57200-000, Brazil

**Keywords:** plastic ingestion, pollution, green turtles, marine debris, bioindicator, monitoring tools

## Abstract

The increase in waste production over recent decades has become a global issue with plastic pollution taking center stage. To respond, we require tools to design and monitor the effectiveness of policies aimed at tackling plastic pollution. However, a key question remains unanswered: “What is the relationship between plastic accumulated in the environment and the plastic ingestion by wildlife?” To address this, we evaluated the relationship between plastic accumulated in the environment and plastic ingested by the green sea turtle (), at regional and local scales. We found a decoupling between plastic in the environment and plastic ingestion at regional and local scale. However, we found a strong relationship when evaluating flexible plastics, where an increase of 1 item/m^2^ on the beach may lead to a more than 100-fold increase in plastic ingestion. A common finding across both scales is the negative correlation between the turtle size and the number of items of plastic ingested by green turtles. Taking advantage of the present results on in Brazilian waters, our work highlights the importance of species’ ecology and environmental plastic abundance for informative monitoring through bioindicator species.

## Graphical abstract

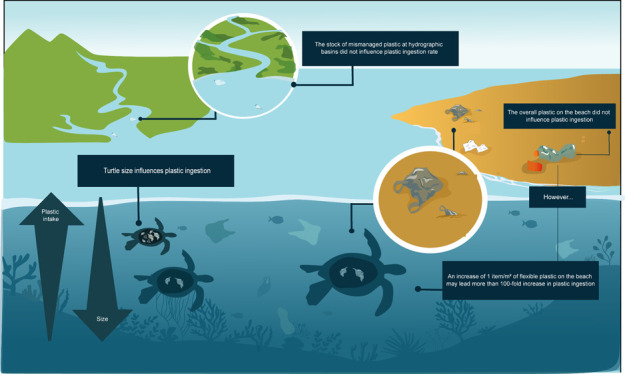

## 1 Introduction

The increase in the production and release of chemical pollutants since the mid 20th-century has become a global problem, such that we are now outside of the safe operating space of the planetary boundary for novel entities.^1^ Plastic pollution stands at the forefront of the transgression of this planetary boundary and worsens^2^ the impacts of the other planetary boundaries.^1,3^ In response to this plastic crisis, several legislations and regulatory initiatives, from local to global, have been proposed and implemented to mitigate plastic pollution.^4,5^

As we approach the adoption of a global treaty to tackle plastic pollution,^6^ a diverse set of tools to monitor and track plastic pollution will be needed to evaluate our progress. Bioindicator species have been proposed and implemented as tools for monitoring plastic pollution.^7–9^ In this context, bioindicators can be understood as species used to assess the health of the environment regarding plastic pollution and may be used to monitor changes over time.^10,11^

Although a positive relationship between the amount of plastic in the environment and the amount ingested by animals is expected, this pattern is poorly understood, with few studies providing conclusive evidence on this issue under real-world circumstances.^12^ A review of the evidence on plastic availability and ingestion found that nearly half of the field studies testing this relationship were inconclusive, while all laboratory-based studies indicated a positive correlation.^12^ These inconclusive results may be influenced by the study design, as plastic availability is one component that influences the risk of plastic ingestion by wildlife, but evolutionary, ecological, and behavioral factors also play a role.^12^ Therefore, designing a study that couples these evolutionary and ecological traits to plastic availability is a challenge. One way to circumvent this is by investigating plastic ingestion across different populations of the same species subjected to different environmental conditions, though only a few studies have adopted this approach.^13^ Understanding the relationship between plastic accumulation in the environment and its ingestion by wildlife is key to designing tools for monitoring plastic pollution. To that end, we tested this relationship at the regional scale (plastics in hydrographic basin) and local scale (plastic collected on beaches where sea turtles stranded) using the green turtle () as a bioindicator. We also assessed debris selectivity by the turtles by testing if the abundance and composition of plastic on the beach differs from those ingested by turtles. Green sea turtles were proposed as a good indicator of plastic pollution in Southern Atlantic^8^ and North Pacific.^7^ Several traits of green turtles qualify them as a bioindicator of plastic pollution: (i) global distribution, enabling their use at a global scale; (ii) their foraging grounds encompass diverse coastal habitats, allowing monitoring of different ecosystems; (iii) high occurrence of plastic ingestion; (iv) they are among the most well-studied species regarding plastic ingestion; therefore, regional historical data may be available for comparison;^14,15^ (v) several sea turtles stranding networks are active worldwide; and (vi) it is charismatic and threatened species (listed as Endangered^16^), which may help to increase public awareness about plastic pollution. Our findings will offer insights as we develop bioindicators of plastic pollution for use in the Global Ocean Observing System (https://goosocean.org/), which provides a comprehensive framework for global ocean monitoring.

## 2 Materials and methods

### 2.1 Green Turtle as a Bioindicator

Green turtles qualify as a good bioindicator of plastic pollution; however, care must be taken when selecting individuals for this purpose. In this context, the intraspecific variation in green turtles’ foraging strategies will affect their encounter rate with plastics, which in turn influences plastic ingestion rate. For the purposes of this study, green turtles were grouped into two main foraging strategies:^12^ (i) Benthic foragers, which feed on benthonic macrophytes such as algae and seagrass, and (ii) water column foragers, which feed on items carried by rivers (e.g., mangrove leaves), jellyfish, or ctenophores from the water column. To determine the foraging strategy, we evaluated the diet items found in the stomach contents of the green turtles. We included only green turtles classified as benthic foragers in our study, as this is the primary foraging strategy employed by green turtles across most of their distribution. If any items suggesting water column foraging were found (e.g., leaves from terrestrial plants or mangrove, jellyfish, ctenophores, or *Sargassum* spp. with air bladders–floating structures), the turtle was excluded from our sample. This procedure was implemented to avoid sampling bias, as plastic ingestion is influenced not only by the level of plastic pollution in the area but also by the foraging ecology of the animals. Including individuals with different foraging ecologies in the same analysis may confound the relationship between environmental plastic availability and ingestion, as the results could be disproportionately affected by the number of animals exhibiting a particular foraging behavior. As most plastics are positively buoyant, green turtles that forage in the water column are expected to have a higher likelihood of ingesting plastic.^17^ Another crucial aspect of using green turtles as bioindicators of plastic pollution is considering their potential mobility. Selecting animals with a more restricted home range is especially important when relating plastic pollution to plastic ingestion at a local scale. Therefore, to ensure a more accurate relationship between plastic availability and green turtle foraging areas, we focused on juvenile green turtles (mean curved carapace length (CCL) = 42.42 cm, SD: 9.13, min–max: 26.1–65). The literature shows that juveniles have smaller home ranges, exhibit relative fidelity to their foraging sites, and their foraging habitats are already established in nearshore reefs.^18,19^ Additionally, unpublished data acquired in the studied area corroborates the relatively small home range of juvenile green turtles (Figure S1). Therefore, this approach of restricting the size of turtles in our study strengthens the link between local plastic availability and their ingestion by turtles. The home range of individuals is especially important when relating plastic pollution to plastic ingestion at a local scale.

From each stranded turtle, we collected the geographical position (latitude and longitude) and measured the CCL. Since the foraging habitats of green turtles are located very close to the shore (ranging from 30 to 2200 m) and the main cause of death in the studied areas are due to fishery interaction,^20,21^ the likelihood of long-distance drifting of carcasses is reduced. Therefore, we considered the geographical location of stranded turtles to be representative of the position of their feeding areas. To evaluate plastic ingestion we collected the entire gastrointestinal tract (GTI). All content of GTs was assessed for macroplastic ingestion (>5 mm), following the protocol described by Matiddi et al.^22^ We separated, sanitized, and dried at room temperature all the anthropogenic items. Each macroplastic item (>5 mm) was measured and categorized according to the type of material (e.g., sheet-like flexible plastic, hard plastic, styrofoam, glass, rubber, nylon/rope, and “other”). The other category included all items that are less commonly found such as metal, cloth, and foam. We counted and weighted (to the nearest 0.01 g) the anthropogenic debris by type to quantify plastic ingestion. To minimize overestimation of the number of ingested debris items, we only counted fragments larger than 5 mm as individual items. Smaller fragments (<5 mm) were presumed to result from the internal fragmentation of larger pieces and were therefore excluded from item counts, though they were included in the total weight. Conversely, plastic pellets (raw materials used in the production of plastic goods) were counted as individual items regardless of their size.

### 2.2 Estimates of Environmental Plastic Pollution

#### 2.2.1 Regional Approach

To quantify the plastic pollution at the regional scale, we retrieved information on mismanaged plastic waste prone to escape (hereinafter referred to as the stock of mismanaged plastic waste) at hydrographic basins estimated by Alencar et al.^23^ along the Brazilian coast. Their estimation takes into consideration, among other things, information on plastic production, socioeconomics, and plastic waste management.^23^ Stocks of mismanaged plastic waste were obtained in tons of plastic per year (https://pactoglobal.org.br/webmapa-bluekeepers). We associated the stock of mismanaged plastic waste for 38 hydrographic basins with plastic ingestion by green turtles (*n* = 329) found stranded during 2010, 2011, 2012, and 2020 in these areas (Figure 1) to build a model to evaluate the relationship between regional plastic pollution and plastic ingestion.

**Figure 1:**
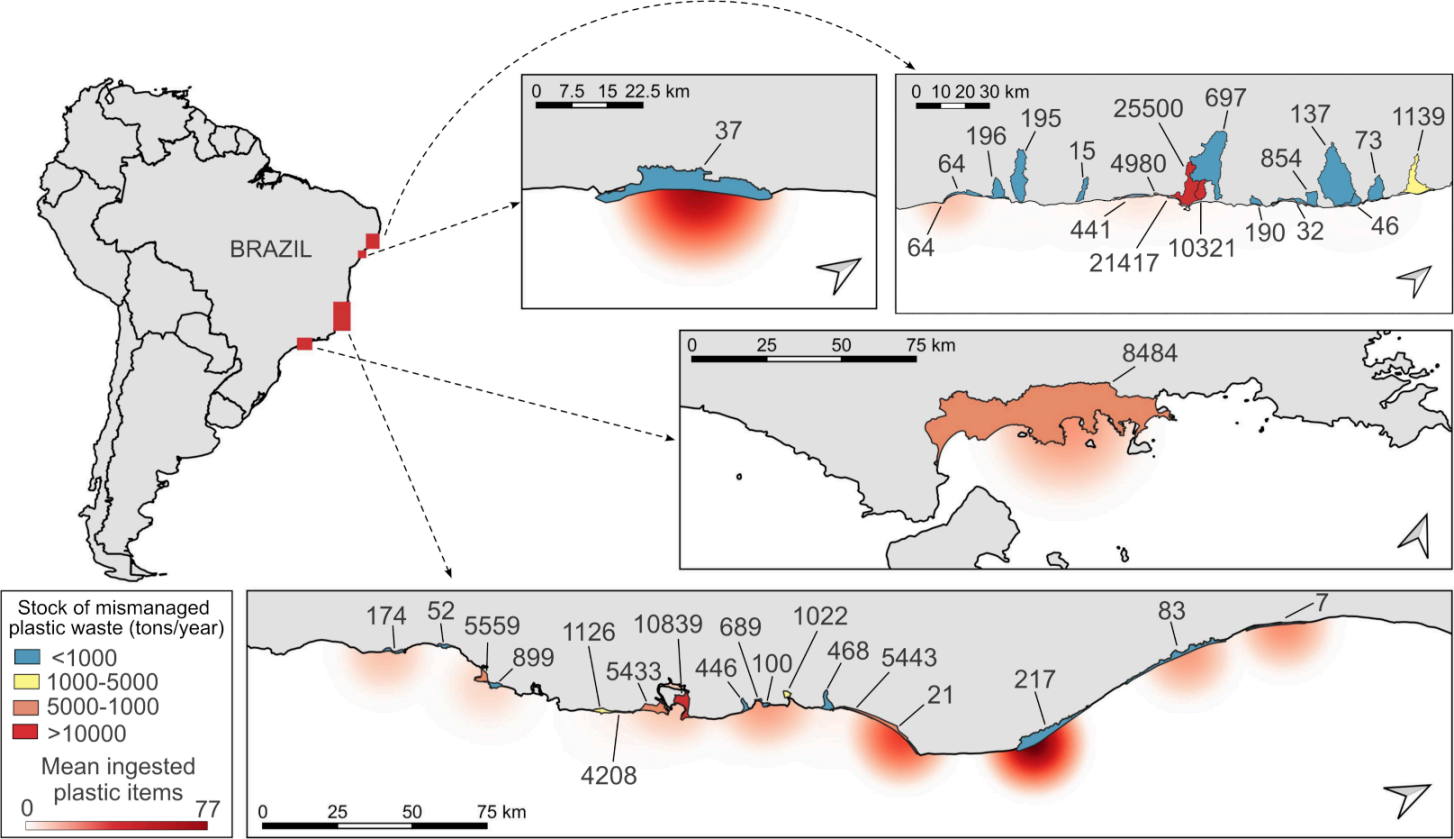
Plastic ingestion and mismanaged plastic waste along the Brazilian coast. The heatmap represents the mean number of plastic items ingested by green turtles () in each hydrographic basin. Numbers adjacent to the basins indicate the amount of mismanaged plastic waste (tons/year).

#### 2.2.2 Local Approach

Beach surveys are the most common methodology to estimate plastic pollution in coastal environments.^4^ This method is considered to provide an index of debris trends in adjacent waters^24^ and has been used as a proxy for plastic availability in the water for green turtles,^21,25^ since their foraging habitats are located very close to the shore (average distance from shore = 1115 m; range: 30–2200 m). Given the limited home range of juvenile green turtles and the proximity of their feeding areas to the shore (see Section 2.1), this strengthens our assumption that debris found on nearby beaches reliably reflects the plastic pollution to which these turtles are exposed in their natural habitats. Therefore, we conducted surveys on the beaches where turtles were found stranded (*n* = 72 beaches) between February and March 2020 and associated this information with plastic ingestion by green turtles (*n* = 144). This narrow survey window was established to collect data on plastic availability under consistent weather conditions across all sites. This strategy was employed to ensure that data from all transects are comparable and not influenced by weather variations, as plastic can be transported to the beaches by strong winds and rainfall. Transect length was measured from the high tide line to the end of the sand strip, while width was fixed to 2 m. All debris readily visible on the sand surface were collected and brought to the laboratory, where they were sanitized and categorized according to the type of material (e.g., sheet-like flexible plastic, hard plastic, styrofoam, glass, rubber, nylon/rope and others) and size (in 5 cm classes; 0–5 cm, 5–10 cm, 10–15 cm, continuing in 5 cm intervals up to >50 cm). Green turtles were collected throughout 2018. The study area for the local approach is similar in terms of human occupation, potential sources of plastics, and green turtles’ foraging habitats. It consists of sandy beaches with coral reefs and small rivers distributed throughout the region. Human occupation is also comparable, primarily consisting of residential areas and commercial enterprises. The degree of urbanization varies between sites, but there are no large-scale industrial activities present. No major events of the removal or introduction of plastics were reported for the locations we examined in this study. Therefore, for comparison purposes, we assumed that plastic availability for potential green turtle ingestion did not significantly fluctuate. To test this assumption, we evaluated data from a long-term plastic pollution monitoring project conducted by our team between 2017 and 2023 (unpublished data set), where 50% of the green turtles were found stranded dead, and we found no significant differences in anthropogenic debris among the years (Table S1 and Figure S2).

### 2.3 Study Design and Statistical Analysis

We addressed the relationship between plastic availability and plastic ingestion by asking three questions, each one with a methodological approach described below.

Does the stock of mismanaged plastic waste at regional scale (hydrographic basins) influence the plastic ingestion?

To answer this question, we attributed a value for plastic availability for each green turtle based on the values of the stock of mismanaged plastic waste (tons/year) in the hydrographic basins where the animal was found stranded. The response variable was the probability of ingestion (0 if the turtle did not ingest plastic and 1 if it did), and the predictor variables were mismanaged plastic waste (tons/year) of the hydrographic basins and the turtles' CCL. The turtle CCL was added to the model due to results of previous studies showing that turtle size has influence in the plastic ingestion.^26–28^ For this test, we used a generalized linear model (GLM) with a quasi-binomial distribution,^29^ which allowed us to control overdispersion.^27^ For these tests, we only used turtles that had ingested at least one plastic item; 22.5% of turtles (27 out of 120 stranded animals) had 1 to 13 pieces of plastic in their gut content. Therefore, we employed a dual analytical approach to evaluate the influence of environmental plastic availability on plastic ingestion by green turtles. First, we modeled the probability of ingestion as a binary response, explicitly including both individuals that ingested plastic (1) and those that did not (0), to assess how environmental plastic levels affect the likelihood of ingestion. In the second stage, we restricted the analysis to only those individuals that had ingested plastic to model the number of items ingested. This distinction was made because the environmental drivers influencing the transition from noningestion (0) to ingestion (1) may differ from those affecting variation within ingestion levels (e.g., from ingesting 1 to 2 items).

Does the amount of plastic on the beach (local scale) influence the plastic ingestion?

To answer this question, we attributed a value for plastic availability for each green turtle based on the mean density of anthropogenic debris of all transects within a 10 km radius buffer around the turtle’s stranding location. Then, we applied GLMs with a quasibinomial distribution. The response variable was the probability of ingestion (0 if the turtle did not ingest plastic and 1 if it did), and the predictor variables were the mean density of plastics in the transects and the turtle CCL. The 10 km buffer criteria were established based on the reported home range of juvenile green turtles.^30–32^ To test the effect of plastic availability on the number of plastic items ingested by green turtles, we used GLM models following the same approach described above. All models were subjected to a stepwise reduction process, retaining only the explanatory variables that contributed significantly to the model’s performance. Model selection was guided by Akaike’s information criterion (AIC), ensuring that the explanatory power of the final model remained comparable to more complex alternatives, thus resulting in the most parsimonious models. Both models were run using a quasi-binomial distribution to avoid overdispersion.^33^

As not all plastics found on the beach surveys are available for ingestion since they may exceed the size that a turtle is capable to ingest, we refined our analysis by focusing only on plastic items that are realistically ingestible by turtles. To define the criteria for selecting these items, we used a data set of 178 green turtles that had ingested plastic along the Brazilian coast. This data set allowed us to identify the main types of plastic ingested by turtles and establish upper size limits for ingestion: sheet-like flexible plastics (<25 cm), hard plastics (<10 cm), and nylon/rope (all sizes). The refined subset of plastic items used to represent plastic availability thus includes only these categories.

Does the relative abundance of plastic on the beach differ from that ingested by turtles?

To address the question regarding the similarity between plastic available in the environment, here represented by the plastic found in the beach, and those ingested by the turtles, we used two data sets: one containing all the debris found on the beach and one containing only the items that were readily available for ingestion by turtles. The relative abundance of each anthropogenic material found on the beach and ingested by turtles were used to build a dissimilarity matrix with Euclidean distances. To test the differences between the relative abundance of plastics on the beach (i.e., potentially ingestible by turtles) and those actually ingested by turtles, we performed a PERMANOVA, using the function “adonis2” of the Vegan package.^34,31^ The response variable was the dissimilarity matrix of the abundances, and the predictor variable was where the plastic was found (deposited on the beach or ingested by turtles). We also applied the “betadisper” function of the Vegan package, followed by a Tukey test, to verify if the dispersion between the points of the two groups was homogeneous. To verify if the variation in the plastics found on the beach affects those ingested by turtles, we applied Mantel tests for the correlation between the dissimilarity matrix of plastics on the beach and those ingested by turtles. All statistical analyses cited above were performed in R 4.3.1 (R Core Team, 2023),^35^ and we used a 95% confidence interval (CI), and for the analysis script, see ESM.

## 3 Results

### 3.1 Regional Scale of Plastic Presence and Ingestion Estimates

The mean value of stock of mismanaged plastic waste was 7450.1 tons/year (SD = 7834.8; range 7–25,500), indicating a high degree of variability among observations (Figure 1). The mean CCL of green turtles was 42.4 cm (SD = 9.12; range 26.1–65). Plastic ingestion was recorded for 54% of 329 turtles, with a total of 2897 plastic items and a mean number of 16.3 items per turtle that ingested debris (SD = 43.9; range: 1–369 items) (Figure 1).

### 3.2 Local Scale of Plastic Presence and Ingestion Estimates

From February to March 2020, we collected 972 individual debris items from 72 transects (24 beaches) spanning approximately 100 km of the Alagoas State coastline, spanning from Barra Grande beach (north) to Ponta Verde beach (south). Mean debris density was 0.73 item/m^2^ (SD = 0.62, range = 0.012–3.40) (Figure 2). Plastic was the most abundant material (60.7%), of which 32.5% were sheet-like flexible plastic, 11.1% hard plastic, 14.9% styrofoam, and 2.1% nylon/rope (Figure 2). Debris within the 0–5 cm and 5–10 cm size classes were the most common comprising 52.5 and 24.58% of the total items, respectively. The mean CCL of green turtles stranded dead in the areas was 49.73 cm (SD = 8.62; range 28.5–65). Plastic ingestion was recorded for 22.5% of the 120 turtles, with a total of 62 plastic items and a mean number of 1.93 items per turtle that ingested debris (SD = 2.27; range: 1–13 items). Sheet-like flexible plastic was the most ingested debris (70.9%), followed by nylon/rope (25.8%; Figure 2).

**Figure 2:**
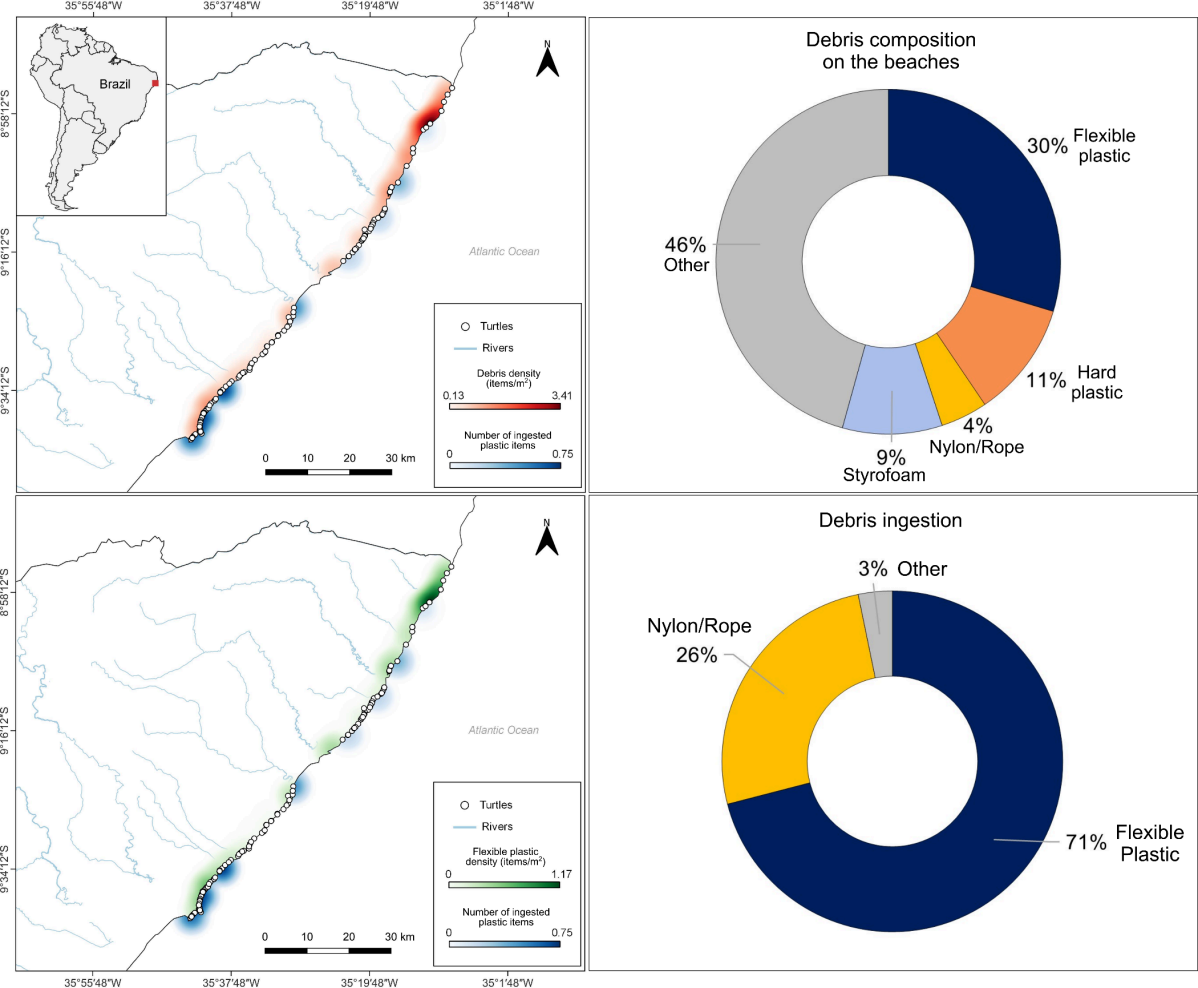
Heatmaps showing the association between the amount of plastic debris (red: all types of plastic; green: flexible plastic) surveyed along the coast of Alagoas State (via transects) and the plastic ingested by green turtles () within the same region (10 km radius buffer). Graphs illustrate the composition of plastic found on beaches (top) and ingested by green turtles (bottom).

### 3.3 Does the stock of mismanaged plastic waste at regional scale (hydrographic basins) influence the plastic ingestion?

The probability of plastic ingestion and the amount of plastic ingested were not influenced by the stock of mismanaged plastic waste at hydrographic basins. The probability of plastic ingestion was only affected by CCL (Table S2) (Figure 3). We observed that for each centimeter in CCL, there is a 5% decrease in the probability of plastic ingestion (Figure 4A). We recovered the same pattern for the number of ingested items, in which for each centimeter in CCL, there is a 0.06 decrease in the number of ingested items (Table S3) (Figure 4B).

**Figure 3:**
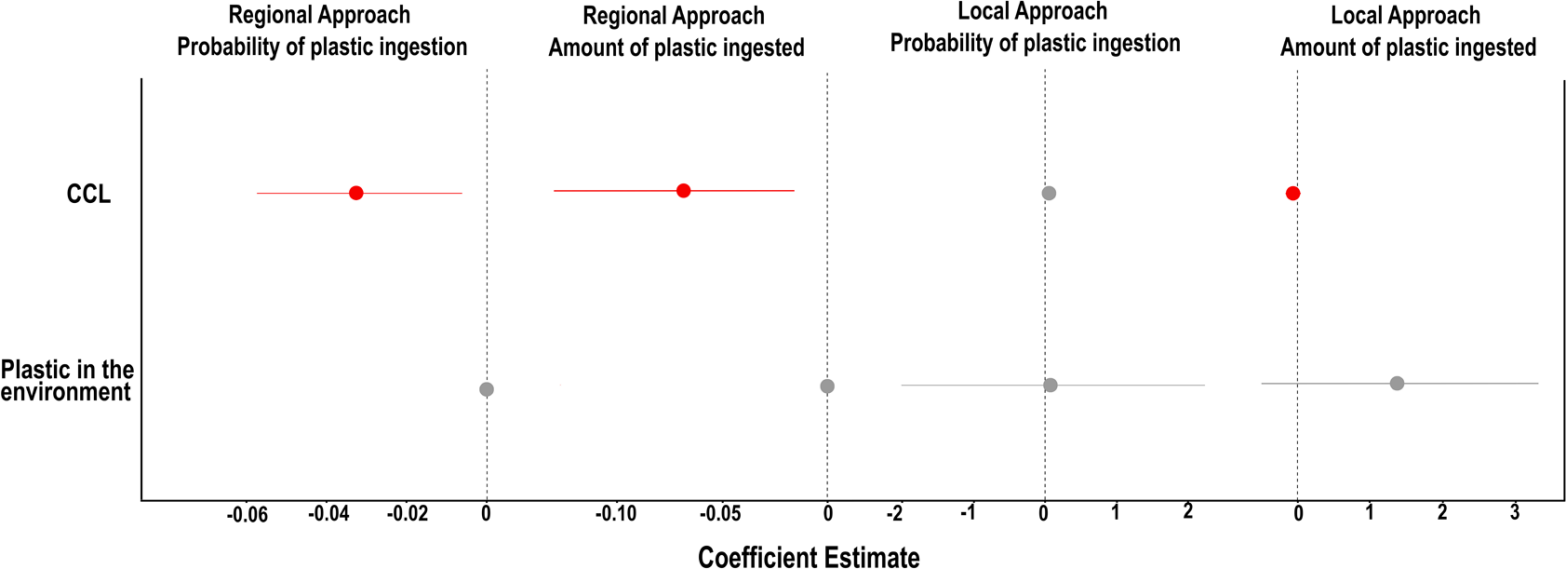
Coefficient estimates for the effects of CCL and environmental plastic availability on the probability of plastic ingestion and the amount of plastic ingested by green turtles () along the Brazilian coast. Analyses were performed on both regional and local spatial scales. The probability of plastic ingestion refers to the likelihood of finding plastic debris in the GTI, while the amount of plastic ingested represents the number of plastic items found within GTIs. Error bars indicate 95% CIs. Red indicates a significant negative effect, and gray indicates no effect.

**Figure 4:**
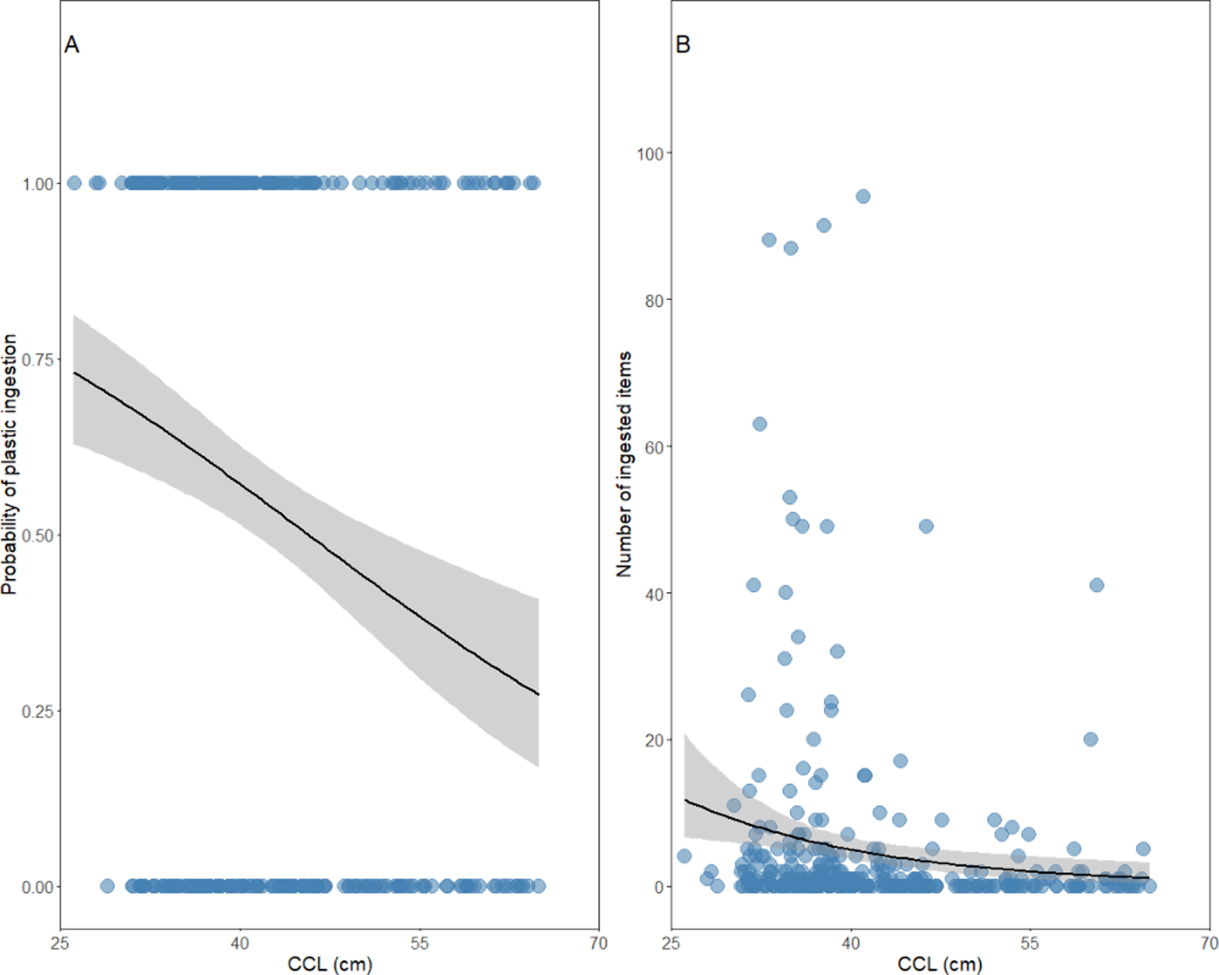
Relationship between the CCL and (A) the probability of plastic ingestion and (B) the number of plastic items ingested by green turtles () along the Brazilian coast. Black lines represent predictions from GLMs and shaded areas indicate 95% CIs.

### 3.4 Does the amount of plastic on the beach (local scale) influence plastic ingestion?

The total plastic density on the beaches and the turtles CCL did not affect the probability of plastic ingestion (d. = 2, *F* = 0.835, *p* = 0.0436) (Figure 3). However, the number of plastic items ingested by each turtle was affected by CCL (Table S4)­(Figure 3), and an increase in 1 cm in CCL decreased the number of ingested plastic items by 0.03 units (Figure 5A). When we considered plastic density on the beach by plastic types, none of them affected the probability of plastic ingestion (df = 5, *F* = 0.4485, *p* = 0.8138). However, some plastic types affected the number of plastic items ingested (Table S5) (Figure 5B). This analysis showed that an increase of one unit of flexible plastic per square meter may cause the number of items ingested by green turtles to increase more than 100 times.

**Figure 5:**
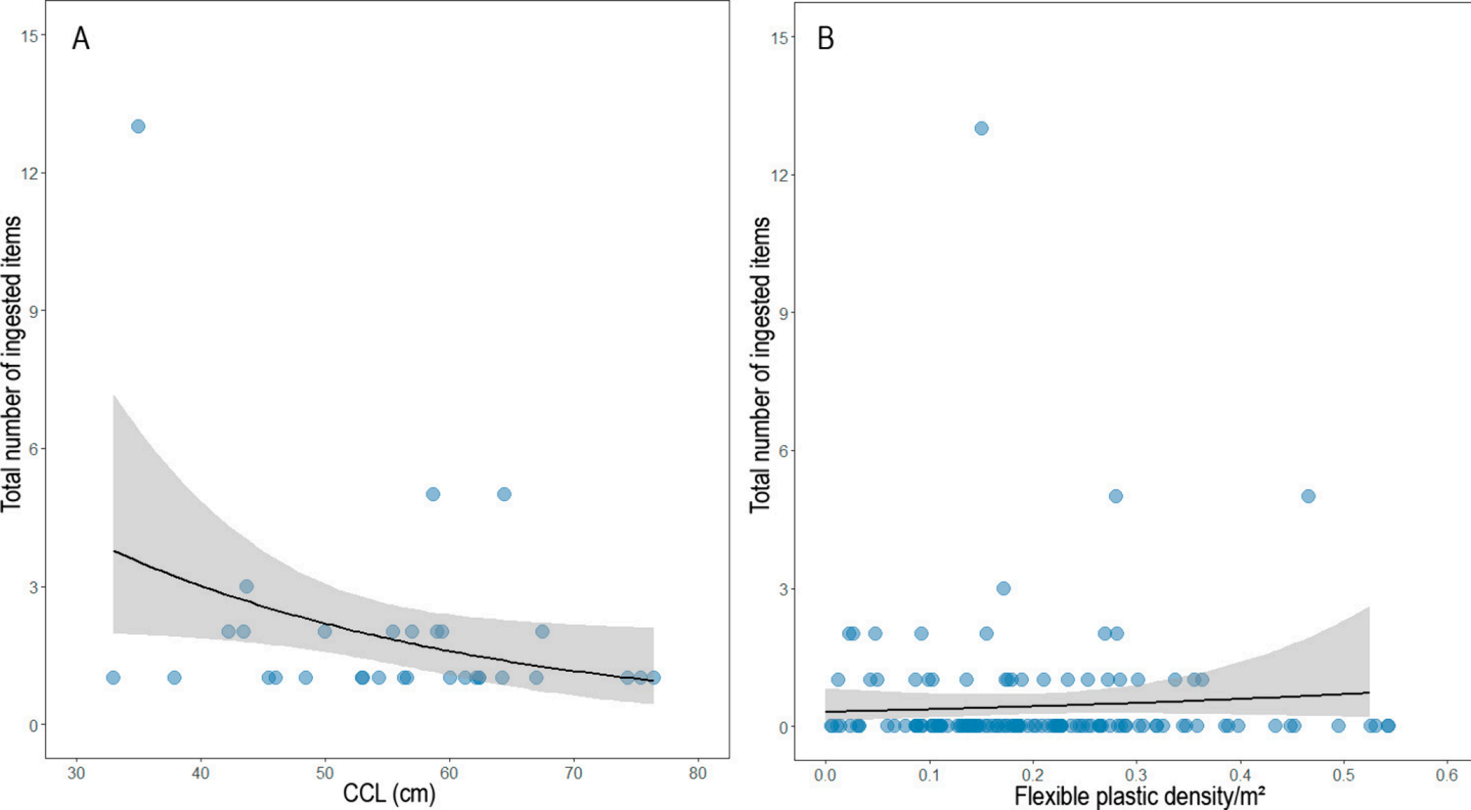
Effects of (A) CCL on the total number of plastic items ingested by stranded green turtles () and (B) the effect of the availability of flexible plastic items on beaches on plastic ingestion by green turtles. The black lines represent predictions from GLMs, and the shaded areas indicate 95% CIs.

### 3.5 Does the abundance and composition of plastic on the beach differ from those ingested by turtles?

Plastic composition on the beaches and those ingested by green turtle were significantly different (d. f. = 1, *R* ^2^ = 0.1951, *F* = 11.635, *p* < 0.001), and the Mantel test provided further support that the variation of the plastic composition on the beaches and in green turtle GTs is not correlated (*r* = 0.071, *p* = 0.1287).

## 4 Discussion

We found no relationship between the stock of mismanaged plastic waste at a regional scale and plastic ingestion (probability of ingestion or the amount of plastic ingested) by green turtles. We observed a similar scenario when evaluating the relationship between plastic accumulated on beaches and plastic ingested by green turtles, as in our local-scale approach. This apparent decoupling between plastic accumulation in the environment and plastic ingestion, at regional and local scales, may have implications for interpreting evaluations of plastic pollution through bioindicators. In this scenario, the decrease in plastic pollution due to the effectiveness of mitigation actions (or the opposite scenario) at regional or local scales may not be captured by the bioindicator. However, this does not imply that monitoring plastic ingestion is ineffective for evaluating the success of actions to tackle plastic pollution. For example, if species have predilections for specific items, as green turtles show a preference for flexible plastics (e.g., plastic bags, food package) in this study, then they may be particularly useful to track that item in the environment. Plastic ingestion provides a better understanding of the impacts of plastic pollution on biodiversity than data limited to environmental accumulation such as beach and ocean surveys.

At the local scale, we were able to refine the relationship between plastic in the environment and plastic ingested by green turtles. We found that an increase of one unit of flexible plastic per square meter on the beach increases plastic ingestion by over 100 times. This finding highlights the importance of using ecologically relevant scenarios regarding plastic availability, as not all plastics in the environment are available for ingestion due to their size or the environment where they accumulate (e.g., surface, water column, or the bottom). Additionally, plastic ingestion is not determined solely by plastic availability but also by evolutionary, ecological, and behavioral factors, such as resemblance between plastic and prey and feeding behavior.^12^ The importance of the foraging behavior, even when evaluating the same species, is also highlighted by our findings that pointed out a negative relationship between animals’ size and plastic ingestion. This relationship is expected for sea turtles^27^ as smaller individuals tend to have a more generalist diet,^36,37^ which may affect the chance of plastic ingestion.^12^ Additionally, we found differences between the composition of plastic accumulated in the environment and those ingested by green turtles, which suggested selective ingestion. This selection toward some type of plastics is probably due to the overlap of cues given by natural foods and certain types of plastics.^12^ The similarities between plastics and food items may be visual, such as color, as reported in fish that ingest more blue particles due to their resemblance to copepod prey^38^ and in green turtles that preferentially ingest certain colors over others.^26^ Shape also plays a significant role, as seen in sea turtles that favor sheet-like plastics over other forms of plastic debris,^39^ and in zooplankton, whose ingestion of plastics is influenced by particle shape.^40^ Chemical cues also play a role, as biofouled plastics produce algal-derived infochemicals that are attractive to sea turtles^41^ and zooplankton.^40^

Beyond the importance of bioindicators for monitoring plastic pollution, our results may also be used to identify areas where green turtles may be at a greater risk of plastic ingestion. Mapping the areas where green turtles face a higher risk of plastic ingestion is key to evaluating threats of pollution at populational level. Alongside climate change, pollution remains as the main knowledge gaps among all threats for sea turtles’ conservation.^42^ Given that even small amounts of plastic can cause health issues and potentially lethal effects for turtles^20,43,44^ and potential impacts of plastic ingestion at the populational level,^45^ our findings suggest that the amount of plastic on beaches could help predict the risk of plastic ingestion for turtles in nearby foraging areas. However, care must be taken when extrapolating our results to other areas, as the risk of plastic ingestion is influenced by an interaction of different drivers.^12^ Additionally, recent studies have proposed juvenile green turtles as good indicators of plastic pollution in the Southern Atlantic^8^ and North Pacific,^7^ and our findings support this. However, we suggest that monitoring designs should focus on the local scale and that local foraging ecology knowledge should be established for better interpretation of the results.

Tools to monitor plastic pollution are key to evaluating the effectiveness of mitigation actions that will take place under the unfolding global agreement to address plastic pollution. Understanding how the bioindicator responds to plastic in the environment is key to determining what kinds of questions can be answered by assessing plastic ingestion by wildlife. Discussions are ongoing regarding the importance of harmonizing methods to monitor the trends and impacts of plastic pollution.^7,13,46^ However, the care in using bioindicators must extend beyond harmonization and recognize the need for robust knowledge of the foraging ecology of the bioindicator. There exists an evolutionary, ecological, and behavioral “filter” between plastic in the environment and plastic ingested by wildlife. The knowledge of the foraging ecology of the species can guide sample designs to minimize the effects of the ecological and behavioral components of the filter. Recognizing the existence of this filter does not imply that bioindicators are of less use than directly measuring plastics in the environment because they can provide a more comprehensive view of the impacts of plastic pollution on biodiversity and for evaluating the outcome of targeted actions against specific debris items favored by bioindicators. Additionally, another key aspect is understanding the home range of the intended bioindicator species. Highly mobile animals cannot be easily linked to local levels of plastic pollution, especially if the species moves across a heterogeneous plastic pollution seascape (or landscape). In such cases, a broader spatial sampling design is required to capture the variability in exposure to plastic pollution and ensure that ingestion data are ecologically meaningful.

## 5 Implications

Species do not typically ingest all types of plastics in the same proportions as they occur in the environment; they are selective, even filter-feeding animals.^47,48^ Additionally, individual traits may influence the amount of plastic ingested. For example, we found that smaller green turtles tend to ingest more plastics. Therefore, when bioindicators are used to monitor plastic pollution, it is crucial to understand the foraging ecology and preferences of the animals involved. We observed a decoupling between plastic pollution and plastic ingestion by green turtles at both regional (plastics accumulated in hydrographic basins) and local (plastics accumulated on beaches) scales. At first glance, this decoupling may appear to undermine the effectiveness of bioindicators. However, we found a strong relationship between the presence of flexible plastics on beaches and plastic ingestion by sea turtles. These findings suggest that once we identify the relationship between plastic in the environment and plastic ingested by bioindicators, they may become powerful tools for monitoring plastic pollution and creating future scenarios to assess its potential impact on a number of other species. In the specific case of green turtles, our findings support their use as bioindicators of plastic pollution. However, we recommend that monitoring efforts be designed based on the species’ foraging and movement ecology, with an emphasis on the local scale to allow for more accurate interpretation of the results.

## Supplementary Material



## Notes

Scripts for statistical analysis and data sets; plastic density (items/m^2^) per transect per year at the beaches (PDF)
