# Supplementary material for: Bioindicators of Plastic Pollution: Insights into the Relationship between Environmental Plastic Abundance and Ingestion by Green Turtles ()
Source: Environ Sci Technol. 2025 Jul 8;59(28):14605–14. doi: 10.1021/acs.est.5c01171 (PMC12288082; doi:10.1021/acs.est.5c01171)
Supplement: Supplementary file 1 [file es5c01171_si_001.pdf]

Support information for
Bioindicators of plastic pollution: the relationship with plastic in the environment and the importance of ecological knowledge

Robson G Santos[a,*], Adriano Carvalho Vasconcelos[a,b], Priscilla Monteiro de Oliveira[a], João Paulo Felix Augusto de Almeida[a], Ingredy Silva[a,], Bruno Stefanis S.P. de Oliveira[b], Matthew S. Savoca[cd] , Guilherme Ramos Demetrio[e]

[a]ECOA LAB, Instituto de Ciências Biológicas e da Saúde, Universidade Federal de Alagoas, Maceió, AL, Brazil

[b]Instituto Biota de Conservação, 57038-770, Maceió, AL, Brazil

[c] Hopkins Marine Station, Stanford University, Pacific Grove, CA, USA

[d] California Marine Sanctuary Foundation, Monterey, CA, USA

[e]Plant Ecology Lab (LEVE), Universidade Federal de Alagoas, Campus Arapiraca, Unidade Educacional Penedo, Penedo, AL, Brasil

*Corresponding author: robson.santos@icbs.ufal.br

Number of pages: 69
Number of tables: 5
Number of figures: 2

Scripts, datasets, Tables and Figures

Documents S1

Scripts:

Local approach

Regional approach

Datasets:

plastic_transects_ingested

plastic_transects_number

community

dataset_novo_semNA

Figure S1

Table S1

Figure S2

Table S2-5

Documents S1

Scripts:

Local approach

Regional approach

Datasets:

plastic_transects_ingested

plastic_transects_number

community

dataset_novo_semNA

Local approach

```
data<-read.table("plastic_transects_ingested.txt", h=T)
summary(data)

library(psych)
describe(data)

plot(data$prob~data$total_density)
m0<-glm(prob~1, family=quasibinomial, data=data)
m1<-glm(prob~total_density, family=quasibinomial, data=data)
anova(m0,m1,test="F")

m0<-glm(prob~1, family=quasibinomial, data=data)
m1<-glm(prob~dens_nylon+dens_styrofoam+dens_flex+dens_hard+dens_other,
family=quasibinomial, data=data)
anova(m0,m1,test="F")

data<-read.table("plastic_transects_number.txt", h=T)
summary(data)

plot(data$Total_items~data$total_density)
m0<-glm(Total_items~1, family=quasipoisson, data=data)
m1<-glm(Total_items~total_density, family=quasipoisson, data=data)
anova(m0,m1,test="F")

m0<-glm(Total_items~1, family=quasipoisson, data=data)
```

```
m1<-
glm(Total_items~dens_nylon+dens_styrofoam+dens_flex+dens_hard+dens_other,
family=quasipoisson, data=data)

anova(m0,m1,test="F")

summary(m1)

m2<-glm(Total_items~dens_styrofoam+dens_flex+dens_hard+dens_other,
family=quasipoisson, data=data)

anova(m1,m2,test="F")

summary(m2)

m3<-glm(Total_items~dens_styrofoam+dens_flex+dens_other,
family=quasipoisson, data=data)

anova(m2,m3,test="F")

summary(m3)

plot(data$Total_items~data$dens_styrofoam)

plot(data$Total_items~data$dens_flex)

exp(-28.3896) - 1

exp(17.3326) - 1

exp(-6.9784) - 1

figd

figa<-ggplot(data, aes(x=dens_flex, y=Total_items)) +

 geom_point(aes(alpha=0.9, size = 0.1), color = "steelblue",

       position = position_jitter(width = 0.09, height = 0)) +

 geom_smooth(method='glm', method.args=list(family="quasipoisson"),
color="black")+

 scale_y_continuous(limits=c(0,15), breaks=(seq(0,15,3))) +

 scale_x_continuous(limits=c(0,0.6), breaks=(seq(0,0.6,0.1))) +

 theme_bw() +

 labs(x = "Flexible plastic density/m2", y="Total number of ingested items") +

 theme(panel.background = element_rect(fill = "white", colour = "black"),

    panel.grid.major = element_blank(), panel.grid.minor = element_blank(),
```

```
    legend.position = "none",
    axis.text = element_text(size = 12),
    axis.title = element_text(size = 14)) +
  annotate(geom="text", x=0, y=15, label="A",
      color="black", size = 6)
figa
figb<-ggplot(data, aes(x=dens_styrofoam, y=Total_items)) +
  geom_point(aes(alpha=0.9, size = 0.1), color = "steelblue",
       position = position_jitter(width = 0.09, height = 0)) +
  geom_smooth(method='glm', method.args=list(family="quasipoisson"),
color="black")+
  scale_y_continuous(limits=c(0,5.5), breaks=(seq(0,5.5,0.5))) +
  scale_x_continuous(limits=c(0,0.25), breaks=(seq(0,0.25,0.05))) +
  theme_bw() +
  labs(x = "Styrofoam density/m2", y="Total number of ingested items") +
  theme(panel.background = element_rect(fill = "white", colour = "black"),
    panel.grid.major = element_blank(), panel.grid.minor = element_blank(),
    legend.position = "none",
    axis.text = element_text(size = 12),
    axis.title = element_text(size = 14)) +
  annotate(geom="text", x=0, y=5.5, label="B",
      color="black", size = 6)
figb
figc<-ggplot(data, aes(x=dens_other, y=Total_items)) +
  geom_point(aes(alpha=0.9, size = 0.1), color = "steelblue",
       position = position_jitter(width = 0.09, height = 0)) +
  geom_smooth(method='glm', method.args=list(family="quasipoisson"),
color="black")+
  scale_y_continuous(limits=c(0,5.5), breaks=(seq(0,5.5,0.5))) +
```

```
 scale_x_continuous(limits=c(0,0.8), breaks=(seq(0,0.8,0.1))) +
 theme_bw() +
 labs(x = "Other plastics density/m2", y="Total number of ingested items") +
 theme(panel.background = element_rect(fill = "white", colour = "black"),
     panel.grid.major = element_blank(), panel.grid.minor = element_blank(),
     legend.position = "none",
     axis.text = element_text(size = 12),
     axis.title = element_text(size = 14)) +
 annotate(geom="text", x=0, y=5.5, label="C",
       color="black", size = 6)

figa+figb+figc

library(ggstatsplot)
ggcoefstats(m3,
        statistic  = "t",
        conf.int   = TRUE,
        conf.level = 0.95,
        xlab       = "Model estimates",
        ylab       = "Term",
        bf.message = FALSE,
        sort       = "ascending",
        only.significant = FALSE)

library(piecewiseSEM)
library(jtools)
library(ggplot2)
library(psych)
```

```
data<-read.table("data_number.txt", h=T)
summary(data)
describe(data)

plot(data$total_Items_ingested)
df1 <- filter(data, data$total_Items_ingested > 0)



plot(data$total_weight_ingested_.g.~data$items_m2_transects)
plot(data$ingestion~data$selected_items_m2_transects)

hist(data$total_weight_ingested_.g.)

#Carregando pacotes que precisaremos para as correla??es
library(Hmisc)
library(corrplot)

#Teste das correla??es entre as vari?veis preditoras. Em geral, correla??es
#significativas acimas de 0.7 demandam a escolha de uma das vari?veis,
#a menos que essas vari?veis tenham significados biol?gicos diferentes

#calculando os r de pearson (vari?veis num?ricas cont?nuas, pearson)
data.cor<-cbind(data$CCC, data$items_m2_transects)
colnames(data.cor) <- c("CCC","items_m2_transects")
View(data.cor)
cor.matrix.var<-cor(data.cor, method = "spearman")
round(cor.matrix.var,2)
```

```
#testando as signific?ncias
cor.test.var<-cor.mtest(cor.matrix.var, conf.level = 0.95)
cor.test.var

#plot das correla??es
corrplot(cor.matrix.var, p.mat=cor.test.var$p, method='circle',
    type='lower', insig='blank', addCoef.col='black',
    number.cex=0.8, order = 'AOE', diag = FALSE)

m1<-glm(data$total_weight_ingested_.g.~data$total_Items_ingested)
summary(m1)
rsquared(m1)

m0<-glm(ingestion~1, family=quasibinomial, data=data)
m1<-glm(ingestion~CCC+items_m2_transects, family=quasibinomial, data=data)
anova(m0,m1,test="F")
summary(m1)

f1<-dwplot(m1,
     vline = geom_vline(
      xintercept = 0,
      colour = "grey60",
      linetype = 2
     ),
) %>% # plot line at zero _behind_coefs
```

```
 relabel_predictors(
  c(CCC = "CCL",
    items_m2_transects = "Environmental available plastic")
 ) +
 theme_bw() +
 theme(panel.grid.major = element_blank(), panel.grid.minor = element_blank()) +
 xlab("Coefficient Estimate") + ylab("") +
 geom_vline(xintercept = 0,
         colour = "grey60",
         linetype = 2)+
 theme(

  legend.position = "none"
 ) +
 scale_colour_grey(start = .3, end = .7)
f1

#N?o houve efeito do CCC e da Densidade de itens por transecto na probabiliade de ingest?o.

m0<-glm(ingestion~1, family=quasibinomial, data=data)
m1<-glm(ingestion~CCC+selected_items_m2_transects, family=quasibinomial, data=data)
anova(m0,m1,test="F")

hist(data$total_weight_ingested_.g.)

m0<-glm(total_weight_ingested_.g.~1, family=quasipoisson, data=data)
```

```
m1<-glm(total_weight_ingested_.g.~CCC+items_m2_transects,
family=quasipoisson, data=data)

anova(m0,m1,test="F")


m0<-glm(total_weight_ingested_.g.~1, family=quasipoisson, data=data)

m1<-glm(total_weight_ingested_.g.~CCC+selected_items_m2_transects,
family=quasipoisson, data=data)

anova(m0,m1,test="F")


hist(data$total_Items_ingested)

m0<-glm(total_Items_ingested~1, family=quasipoisson, data=data)

m1<-glm(total_Items_ingested~CCC+items_m2_transects, family=quasipoisson,
data=data)

anova(m0,m1,test="F")

summary(m1)


m0<-glm(total_Items_ingested~1, family=quasipoisson, data=data)

m1<-glm(total_Items_ingested~CCC+selected_items_m2_transects,
family=quasipoisson, data=data)

anova(m0,m1,test="F")

summary(m1)


data<-read.table("data_number_nozero.txt", h=T)

summary(data)

SD(data$CCC)

m0<-glm(total_weight_ingested_.g.~1, family=quasipoisson, data=data)
```

```
m1<-glm(total_weight_ingested_.g.~CCC+items_m2_transects,
family=quasipoisson, data=data)

anova(m0,m1,test="F")




m0<-glm(total_weight_ingested_.g.~1, family=quasipoisson, data=data)

m1<-glm(total_weight_ingested_.g.~CCC+selected_items_m2_transects,
family=quasipoisson, data=data)

anova(m0,m1,test="F")


summary(m1)


hist(data$total_Items_ingested)

m0<-glm(total_Items_ingested~1, family=quasipoisson, data=data)

m1<-glm(total_Items_ingested~CCC+items_m2_transects, family=quasipoisson,
data=data)

anova(m0,m1,test="F")


summary(m1)


m2<-glm(total_Items_ingested~CCC, family=quasipoisson, data=data)

anova(m1,m2,test="F")

summary(m2)



exp(-0.0318) - 1
```

```
m3<-glm(total_Items_ingested~CCC+selected_items_m2_transects,
family=quasipoisson, data=data)
summary(m3)


m2<-glm(total_Items_ingested~CCC, family=quasipoisson, data=data)
summary(m2)


figd<-ggplot(data, aes(x=CCC, y=total_Items_ingested)) +
 geom_point(aes(alpha=0.9, size = 0.1), color = "steelblue",
       position = position_jitter(width = 0.09, height = 0)) +
 geom_smooth(method='glm', method.args=list(family="quasipoisson"),
color="black")+
 scale_y_continuous(limits=c(0,15), breaks=(seq(0,15,3))) +
 scale_x_continuous(limits=c(30,80), breaks=(seq(30,80,10))) +
 theme_bw() +
 labs(x = "CCC (cm)", y="Total number of ingested items") +
 theme(panel.background = element_rect(fill = "white", colour = "black"),
    panel.grid.major = element_blank(), panel.grid.minor = element_blank(),
    legend.position = "none",
    axis.text = element_text(size = 12),
    axis.title = element_text(size = 14)) +
 annotate(geom="text", x=30, y=15, label="B",
      color="black", size = 6)
figd


figa+figd
library(dotwhisker)
library(dplyr)
g1<-dwplot(m1,
```

```
    vline = geom_vline(
      xintercept = 0,
      colour = "grey60",
      linetype = 2
    ),
    ) %>% # plot line at zero _behind_coefs
relabel_predictors(
  c(CCC = "CCL",
    items_m2_transects = "Environmental available plastic")
) +
theme_bw() +
theme(panel.grid.major = element_blank(), panel.grid.minor = element_blank()) +
xlab("Coefficient Estimate") + ylab("") +
geom_vline(xintercept = 0,
           colour = "grey60",
           linetype = 2)+
theme(

  legend.position = "none"
) +
scale_colour_grey(start = .3, end = .7)
```

```
m3<-glm(total_Items_ingested~CCC+selected_items_m2_transects,
family=quasipoisson, data=data)
```

```
summary(m2)
exp(-0.03417) - 1
exp(2.36175) - 1

g2<-dwplot(m3,
      vline = geom_vline(
        xintercept = 0,
        colour = "grey60",
        linetype = 2
      ),
) %>% # plot line at zero _behind_coefs
  relabel_predictors(
    c(CCC = "CCL",
      selected_items_m2_transects = "Number of selected plastic items/m?")
  ) +
  theme_bw() +
  theme(panel.grid.major = element_blank(), panel.grid.minor = element_blank()) +
  xlab("Coefficient Estimate") + ylab("") +
  geom_vline(xintercept = 0,
            colour = "grey60",
            linetype = 2)+
  theme(

    legend.position = "none"
  ) +
  scale_colour_grey(start = .3, end = .7)
```

```
plot(data$total_Items_ingested~data$selected_items_m2_transects)

library(cowplot)
p1<-plot_grid(g1, g2, labels = "AUTO")
p1

ggsave2(
  filename="effect_size",
  plot = p1,
  device = "pdf",
  width = 30,
  height = 10,
  units = "cm",
  dpi = 300)

ggsave2(
  filename="effect_size_eps",
  plot = p1,
  device = "eps",
  width = 30,
  height = 10,
  units = "cm",
  dpi = 300)

data<-read.table("community.txt", h=T)
summary(data)
data$Turtle_ID<-as.factor(data$Turtle_ID)
data$area<-as.factor(data$area)
```

```
summary(data)


library(vegan)

dij<- vegdist(data[2:5], method = 'euclidean')

adonis2(dij~area, permutations = 10000, data=data)

Area<-as.factor(data[,7])

View(data)

mds.total<-metaMDS(dij)


data.scores <- as.data.frame(scores(mds.total))

data.scores$site <- rownames(data.scores)

data.scores$Area <- Area

head(data.scores)


species.scores <- as.data.frame(scores(mds.total, "species"))

species.scores$species <- rownames(species.scores)

mod.total <- betadisper(dij, Area)

TukeyHSD(mod.total)

disper1<-plot(mod.total)


g1<-ggplot(data=data.scores) +

  geom_point(aes(x=NMDS1,y=NMDS2,shape=Area,colour=Area, size=Area))+

  stat_ellipse(aes(x=NMDS1,y=NMDS2, colour=Area),level = 0.95) +

  scale_color_manual(values=c('darkorange3','dodgerblue4')) +

  scale_shape_manual(values=c(16,15)) +

  scale_size_manual(values=c(3,3)) +

  labs(color = "Sampled compartment", shape = "Sampled compartment", size =
"Sampled compartment") +
```

```
 theme_bw()+
 theme(panel.grid.major = element_blank(),
      panel.grid.minor = element_blank(),
      legend.position = "inside",
      legend.position.inside = c(0.1,0.15),
      legend.title=element_text(size=16),
      legend.text = element_text(size=14))

g1

library(permute)
library(lattice)

praia <- data[1:25,2:5]
tartarugas <- data[26:50,2:5]
dist.praia <- vegdist(praia, method = "euclidean")
dist.tartarugas <- vegdist(tartarugas, method = "euclidean")
mantel(dist.praia, dist.tartarugas, permutations = 10000)
```

Regional approach

```
data<-read.table("dataset_novo_semNA.txt", h=T)
summary(data)
data$Beach<-as.factor(data$Beach)
data$Species<-as.factor(data$Species)
data$EPRPEclassification<-as.factor(data$EPRPEclassification)
data$litter_leakage<-as.factor(data$litter_leakage)
data$State<-as.factor(data$State)
```

```
colnames(data)[3]<-"CCC"

summary(data)

library(patchwork)

library(ggplot2)

library(psych)

data.description<-describe(data)

write.csv(data.description, file = "dataset_semNA.csv", row.names = TRUE)


summary(data)


plot(data$EPRPE~data$Beach)

m0<-glm(EPRPE~1, family=poisson, data=data)

m1<-glm(EPRPE~Beach, family=poisson, data=data)

anova(m0,m1,test="F")

summary(m1)


plot(data$Plastic_ingestion~data$EPRPE)

plot(data$N_Items_ingested~data$EPRPE)

plot(data$Weight_ingested~data$EPRPE)


library(glmmTMB)

library(margins)


plot(data$EPRPE~data$State)


#Probabilidade de ingestão----

m0<-glm(Plastic_ingestion ~ 1, family=quasibinomial, data=data,
na.action="na.exclude")
```

```
m1<-glm(Plastic_ingestion ~ CCC + EPRPE, family=quasibinomial, data=data,
na.action="na.exclude")

anova(m0,m1,test="F")

summary(m1)

m2<-glm(Plastic_ingestion ~ CCL, family=quasibinomial, data=data,
na.action="na.exclude")

anova(m1,m2,test="F")

summary(m2)


colnames(data)[7]<-"Environmental available plastic"

colnames(m1)

f2<-dwplot(m1,

      vline = geom_vline(

       xintercept = 0,

       colour = "grey60",

       linetype = 2

      ),

) %>% # plot line at zero _behind_coefs

 +

 theme_bw() +

 theme(panel.grid.major = element_blank(), panel.grid.minor = element_blank()) +

 xlab("Coefficient Estimate") + ylab("") +

 geom_vline(xintercept = 0,

       colour = "grey60",

       linetype = 2)+

 theme(


  legend.position = "none"

 ) +
```

```
  scale_colour_grey(start = .3, end = .7)
f2


f1+f2
exp(-0.05099)


plot(data$Plastic_ingestion~data$CCL)


figa<-ggplot(data, aes(x=CCL, y=Plastic_ingestion)) +
  geom_point(aes(alpha=0.9, size = 0.1), color = "steelblue",
             position = position_jitter(width = 0.09, height = 0)) +
  geom_smooth(method='glm', method.args=list(family="quasibinomial"),
color="black")+
  scale_y_continuous(limits=c(0,1.2), breaks=(seq(0,1,0.25))) +
  scale_x_continuous(expand=c(0,0),limits=c(25,70), breaks=(seq(25,70,15))) +
  theme_bw() +
  labs(x = "CCL (cm)", y="Probability of plastic ingestion") +
  theme(panel.background = element_rect(fill = "white", colour = "black"),
        panel.grid.major = element_blank(), panel.grid.minor = element_blank(),
        legend.position = "none",
        axis.text = element_text(size = 12),
        axis.title = element_text(size = 14)) +
  annotate(geom="text", x=26, y=1.20, label="A",
           color="black", size = 6)


m0<-glm(data$N_Items_ingested~1, family=quasipoisson, data=data)
m1<-glm(data$N_Items_ingested~ CCL + EPRPE, family=quasipoisson, data=data)
anova(m0,m1,test="Chi")
```

```
summary(m1)

m2<-glm(data$N_Items_ingested~ CCL, family=quasipoisson, data=data)

anova(m1,m2,test="Chi")

summary(m2)

exp(-0.06529) - 1

datafigb<-data[-c(97, 328,98,37,184, 27),]


figb<-ggplot(datafigb, aes(x=CCL, y=N_Items_ingested)) +

  geom_point(aes(alpha=0.9, size = 0.1), color = "steelblue",

          position = position_jitter(width = 0.09, height = 0)) +

  geom_smooth(method='glm', method.args=list(family="quasipoisson"),
color="black")+

  scale_y_continuous(limits=c(0,115), breaks=(seq(0,100,20))) +

  scale_x_continuous(expand=c(0,0),limits=c(25,70), breaks=(seq(25,70,15))) +

  theme_bw() +

  labs(x = "CCL (cm)", y="Number of ingested items") +

  theme(panel.background = element_rect(fill = "white", colour = "black"),

      panel.grid.major = element_blank(), panel.grid.minor = element_blank(),

      legend.position = "none",

      axis.text = element_text(size = 12),

      axis.title = element_text(size = 14)) +

  annotate(geom="text", x=26, y=115, label="B",

        color="black", size = 6)


figa+figb


plot(data$Plastic_ingestion~data$EPRPE)

hist(data$EPRPE)
```

```
p2<-dwplot(m1,
        vline = geom_vline(
          xintercept = 0,
          colour = "grey60",
          linetype = 2
        ),
) %>% # plot line at zero _behind_coefs
  relabel_predictors(
    c(CCL = "CCL",
      EPRPE = "Environmental available plastic")
  ) +
  theme_bw() +
  theme(panel.grid.major = element_blank(), panel.grid.minor = element_blank()) +
  xlab("Coefficient Estimate") + ylab("") +
  geom_vline(xintercept = 0,
          colour = "grey60",
          linetype = 2)+
  theme(

    legend.position = "none"
  ) +
  scale_colour_grey(start = .3, end = .7)
p2

plot(data$total_Items_ingested~data$selected_items_m2_transects)
```

```
library(cowplot)
g1<-plot_grid(g1, p2, labels = "AUTO")
g1

m0<-glm(data$Weight_ingested~1, family=quasipoisson, data=data)
m1<-glm(data$Weight_ingested~ CCL + EPRPE, family=quasipoisson, data=data)
anova(m0,m1,test="Chi")
```

DATASET: plastic_transects_ingested

Turtle_ID prob Lat Long N_transects Total_Transect_area_m2 dens_nylon porc_nylon
transect_Nylon/rope dens_styrofoam porc_styrofoam transect_Styrofoam dens_flex porc_flex
transect_Flex dens_hard porc_hard transect_Hard dens_other porc_other transect_Others
total_density transect_Total_items
T1T107 0 -9.140839 -35.287237 9 221.2 0.004520796 0.007874016 1
0.158227848 0.275590551 35 0.2079566 0.362204724 46 0.099457505 0.173228346
22 0.1039783 0.181102362 23 0.574141049 127
T1T110 0 -8.944311 -35.169845 15 215.66 0.027821571 0.034482759 6
0.180840211 0.224137931 39 0.324584995 0.402298851 70 0.06028007 0.074712644
13 0.213298711 0.264367816 46 0.806825559 174
T1T116 0 -9.045179 -35.236564 8 179.6 0.022271715 0.022857143 4
0.13363029 0.137142857 24 0.267260579 0.274285714 48 0.139198218 0.142857143
25 0.412026726 0.422857143 74 0.974387528 175
T1T117 0 -9.045179 -35.236564 8 179.6 0.022271715 0.022857143 4
0.13363029 0.137142857 24 0.267260579 0.274285714 48 0.139198218 0.142857143
25 0.412026726 0.422857143 74 0.974387528 175
T1T119 0 -9.132235 -35.285097 6 129.8 0.00770416 0.015151515 1
0.154083205 0.303030303 20 0.092449923 0.181818182 12 0.138674884 0.272727273
18 0.115562404 0.227272727 15 0.508474576 66
T1T121 0 -9.083247 -35.250767 9 250.6 0.023942538 0.034090909 6
0.127693536 0.181818182 32 0.159616919 0.227272727 40 0.11971269 0.170454545
30 0.271348763 0.386363636 68 0.702314445 176
T1T122 0 -8.914256 -35.152264 7 128.1 0.023419204 0.035714286 3
0.148321624 0.226190476 19 0.218579235 0.333333333 28 0.06245121 0.095238095
8 0.202966432 0.30952381 26 0.655737705 84
T1T123 0 -9.004481 -35.214375 9 118.06 0.033881077 0.024539877 4
0.152464848 0.110429448 18 0.5251567 0.380368098 62 0.143994579 0.104294479
17 0.5251567 0.380368098 62 1.380653905 163
T1T48 0 -8.963819 -35.175211 15 200.06 0.034989503 0.044585987 7
0.139958013 0.178343949 28 0.32990103 0.420382166 66 0.044986504 0.057324841
9 0.234929521 0.299363057 47 0.784764571 157
T1T7-20 0 -8.928337 -35.162035 12 178.3 0.028042625 0.042735043 5
0.168255749 0.256410256 30 0.201906898 0.307692308 36 0.0448682 0.068376068
8 0.213123948 0.324786325 38 0.65619742 117
T1T76 0 -9.129348 -35.283353 9 221.2 0.004520796 0.007874016 1
0.158227848 0.275590551 35 0.2079566 0.362204724 46 0.099457505 0.173228346
22 0.1039783 0.181102362 23 0.574141049 127
T1T79 0 -9.129348 -35.283352 9 221.2 0.004520796 0.007874016 1

0.158227848 0.275590551 35 0.2079566 0.362204724 46 0.099457505 0.173228346
22 0.1039783 0.181102362 23 0.574141049 127
T1T85 0 -8.999937 -35.208354 9 118.06 0.033881077 0.024539877 4
0.152464848 0.110429448 18 0.5251567 0.380368098 62 0.143994579 0.104294479
17 0.5251567 0.380368098 62 1.380653905 163
T1T91 0 -9.054997 -35.237124 6 170.6 0.029308324 0.035211268 5
0.117233294 0.14084507 20 0.193434936 0.232394366 33 0.134818288 0.161971831
23 0.357561547 0.429577465 61 0.832356389 142
T1T9-20 0 -8.991589 -35.199852 14 168.28 0.035654861 0.030612245 6
0.172331828 0.147959184 29 0.415973378 0.357142857 70 0.101022106 0.086734694
17 0.439743285 0.37755102 74 1.164725458 196
T1T98 0 -9.045149 -35.236929 8 179.6 0.033407572 0.028571429 6
0.122494432 0.104761905 22 0.278396437 0.238095238 50 0.183741648 0.157142857
33 0.551224944 0.471428571 99 1.169265033 210
T2T100 0 -9.224858 -35.332822 6 116.6 0.034305317 0.05 4 0.051457976
0.075 6 0.180102916 0.2625 21 0.025728988 0.0375 3 0.394511149 0.575 46
0.686106346 80
T2T103 0 -9.208261 -35.325023 6 116.6 0.034305317 0.05 4 0.051457976
0.075 6 0.180102916 0.2625 21 0.025728988 0.0375 3 0.394511149 0.575 46
0.686106346 80
T2T104 0 -9.179131 -35.298745 6 79.6 0.037688442 0.056603774 3
0.125628141 0.188679245 10 0.125628141 0.188679245 10 0.150753769 0.226415094
12 0.226130653 0.339622642 18 0.665829146 53
T2T105 0 -9.229095 -35.340251 6 116.6 0.034305317 0.05 4 0.051457976
0.075 6 0.180102916 0.2625 21 0.025728988 0.0375 3 0.394511149 0.575 46
0.686106346 80
T2T112 0 -9.270559 -35.367935 5 109.4 0.036563071 0.050632911 4
0.054844607 0.075949367 6 0.191956124 0.265822785 21 0.027422303 0.037974684
3 0.411334552 0.569620253 45 0.722120658 79
T2T1-20 0 -9.26485 -35.36348 6 139 0.007194245 0.0125 1 0.043165468
0.075 6 0.172661871 0.3 24 0.014388489 0.025 2 0.338129496 0.5875 47
0.575539568 80
T2T121 0 -9.200526 -35.320901 3 29.8 0.100671141 0.142857143 3
0.067114094 0.095238095 2 0.167785235 0.238095238 5 0.033557047 0.047619048
1 0.33557047 0.476190476 10 0.704697987 21
T2T123 0 -9.199497 -35.320291 3 29.8 0.100671141 0.142857143 3
0.067114094 0.095238095 2 0.167785235 0.238095238 5 0.033557047 0.047619048
1 0.33557047 0.476190476 10 0.704697987 21

T2T16-20	0	-9.119172	-35.269427	12	326	0.009202454	0.014851485	3
0.153374233	0.247524752	50	0.17791411	0.287128713	58	0.104294479	0.168316832
34	0.174846626	0.282178218	57	0.619631902	202
T2T18-20	0	-9.279354	-35.376816	6	139	0.007194245	0.0125	1
0.043165468	0.075	6	0.172661871	0.3	24	0.014388489	0.025	2	0.338129496
0.5875	47	0.575539568	80
T2T2-20	0	-9.20967	-35.32439	6	116.6	0.034305317	0.05	4	0.051457976
0.075	6	0.180102916	0.2625	21	0.025728988	0.0375	3	0.394511149	0.575	46
0.686106346	80
T2T26	0	-9.215828	-35.327898	6	116.6	0.034305317	0.05	4	0.051457976
0.075	6	0.180102916	0.2625	21	0.025728988	0.0375	3	0.394511149	0.575	46
0.686106346	80
T2T3-20	0	-9.20646	-35.32382	5	58.2	0.068728522	0.083333333	4
0.068728522	0.083333333	4	0.223367698	0.270833333	13	0.034364261	0.041666667
2	0.429553265	0.520833333	25	0.824742268	48
T2T33	0	-9.242848	-35.346781	3	86.8	0.011520737	0.016949153	1
0.046082949	0.06779661	4	0.184331797	0.271186441	16	0.023041475	0.033898305
2	0.414746544	0.610169492	36	0.679723502	59
T2T37	0	-9.23957	-35.34407	3	86.8	0.011520737	0.016949153	1
0.046082949	0.06779661	4	0.184331797	0.271186441	16	0.023041475	0.033898305
2	0.414746544	0.610169492	36	0.679723502	59
T2T38	0	-9.18535	-35.301591	6	79.6	0.037688442	0.056603774	3
0.125628141	0.188679245	10	0.125628141	0.188679245	10	0.150753769	0.226415094
12	0.226130653	0.339622642	18	0.665829146	53
T2T41	0	-9.221212	-35.341857	6	116.6	0.034305317	0.05	4	0.051457976
0.075	6	0.180102916	0.2625	21	0.025728988	0.0375	3	0.394511149	0.575	46
0.686106346	80
T2T49	0	-9.19589	-35.312568	3	29.8	0.100671141	0.142857143	3
0.067114094	0.095238095	2	0.167785235	0.238095238	5	0.033557047	0.047619048
1	0.33557047	0.476190476	10	0.704697987	21
T2T55	0	-9.237506	-35.343066	3	86.8	0.011520737	0.016949153	1
0.046082949	0.06779661	4	0.184331797	0.271186441	16	0.023041475	0.033898305
2	0.414746544	0.610169492	36	0.679723502	59
T2T6-20	0	-9.23426	-35.34106	3	86.8	0.011520737	0.016949153	1
0.046082949	0.06779661	4	0.184331797	0.271186441	16	0.023041475	0.033898305
2	0.414746544	0.610169492	36	0.679723502	59
T2T63	0	-9.216961	-35.328175	6	166.6	0.024009604	0.05	4	0.036014406
0.075	6	0.12605042	0.2625	21	0.018007203	0.0375	3	0.276110444	0.575	46

0.480192077 80
T2T7-20 0 -9.23545 -35.34208 3 86.8 0.011520737 0.016949153 1
0.046082949 0.06779661 4 0.184331797 0.271186441 16 0.023041475 0.033898305
2 0.414746544 0.610169492 36 0.679723502 59
T2T81 0 -9.188453 -335.303289 6 79.6 0.037688442 0.056603774 3
0.125628141 0.188679245 10 0.125628141 0.188679245 10 0.150753769 0.226415094
12 0.226130653 0.339622642 18 0.665829146 53
T2T8-20 0 -9.24274 -35.34645 3 86.8 0.011520737 0.016949153 1
0.046082949 0.06779661 4 0.184331797 0.271186441 16 0.023041475 0.033898305
2 0.414746544 0.610169492 36 0.679723502 59
T2T85 0 -9.261948 -35.360342 4 102.2 0.009784736 0.016393443 1
0.039138943 0.06557377 4 0.176125245 0.295081967 18 0.019569472 0.032786885
2 0.352250489 0.590163934 36 0.596868885 61
T2T88 0 -9.175941 -35.297863 7 109.2 0.027472527 0.044776119 3
0.10989011 0.179104478 12 0.192307692 0.313432836 21 0.119047619 0.194029851
13 0.164835165 0.268656716 18 0.613553114 67
T2T9-20 0 -9.27877 -35.37619 6 139 0.007194245 0.0125 1 0.043165468
0.075 6 0.172661871 0.3 24 0.014388489 0.025 2 0.338129496 0.5875 47
0.575539568 80
T2T96 0 -9.219314 -35.328873 6 166.6 0.024009604 0.05 4 0.036014406
0.075 6 0.12605042 0.2625 21 0.018007203 0.0375 3 0.276110444 0.575 46
0.480192077 80
T2T97 0 -9.264018 -35.362634 6 139 0.007194245 0.0125 1 0.043165468
0.075 6 0.172661871 0.3 24 0.014388489 0.025 2 0.338129496 0.5875 47
0.575539568 80
T3T108 0 -9.450684 -35.529718 3 65.5 0.015267176 0.043478261 1
0.061068702 0.173913043 4 0.076335878 0.217391304 5 0 0 0
0.198473282 0.565217391 13 0.351145038 23
T3T109 0 -9.428528 -35.5064 3 88 0.011363636 0.011494253 1
0.159090909 0.16091954 14 0.352272727 0.356321839 31 0.136363636 0.137931034
12 0.329545455 0.333333333 29 0.988636364 87
T3T117 0 -9.450042 -35.529725 3 65.5 0.015267176 0.043478261 1
0.061068702 0.173913043 4 0.076335878 0.217391304 5 0 0 0
0.198473282 0.565217391 13 0.351145038 23
T3T12 0 -9.474221 -35.553936 6 128.2 0.007800312 0.023809524 1
0.03900156 0.119047619 5 0.070202808 0.214285714 9 0.007800312 0.023809524
1 0.202808112 0.619047619 26 0.327613105 42
T3T121 0 -9.474288 -35.553246 6 128.2 0.007800312 0.023809524 1

0.03900156 0.119047619 5 0.070202808 0.214285714 9 0.007800312 0.023809524
1 0.202808112 0.619047619 26 0.327613105 42
T3T125 0 -9.465833333 -35.54333333 6 128.2 0.007800312 0.023809524 1
0.03900156 0.119047619 5 0.070202808 0.214285714 9 0.007800312 0.023809524
1 0.202808112 0.619047619 26 0.327613105 42
T3T37 0 -9.431846 -35.509543 4 113.6 0.017605634 0.021505376 2
0.132042254 0.161290323 15 0.281690141 0.344086022 32 0.105633803 0.129032258
12 0.281690141 0.344086022 32 0.818661972 93
T3T50 0 -9.400441 -35.496373 3 88 0.011363636 0.011494253 1
0.159090909 0.16091954 14 0.352272727 0.356321839 31 0.136363636 0.137931034
12 0.329545455 0.333333333 29 0.988636364 87
T3T65 0 -9.400327 -35.496336 3 88 0.011363636 0.011494253 1
0.159090909 0.16091954 14 0.352272727 0.356321839 31 0.136363636 0.137931034
12 0.329545455 0.333333333 29 0.988636364 87
T3T73 0 -9.411194 -35.506696 3 88 0.011363636 0.011494253 1
0.159090909 0.16091954 14 0.352272727 0.356321839 31 0.136363636 0.137931034
12 0.329545455 0.333333333 29 0.988636364 87
T3T79 0 -9.411206 -35.506659 3 88 0.011363636 0.011494253 1
0.159090909 0.16091954 14 0.352272727 0.356321839 31 0.136363636 0.137931034
12 0.329545455 0.333333333 29 0.988636364 87
T4T112 0 -9.541113 -35.61442 6 75.4 0 0 0 0.053050398
0.210526316 4 0.092838196 0.368421053 7 0.026525199 0.105263158 2
0.079575597 0.315789474 6 0.25198939 19
T4T124 0 -9.620266 -35.691299 9 241.4 0 0 0 0.037282519
0.064285714 9 0.182270091 0.314285714 44 0.057995029 0.1 14 0.302402651
0.521428571 73 0.57995029 140
T4T131 0 -9.61185 -35.68501 8 217.2 0 0 0 0.036832413
0.06557377 8 0.174953959 0.31147541 38 0.064456722 0.114754098 14
0.285451197 0.508196721 62 0.561694291 122
T4T137 0 -9.575048 -35.655268 6 126 0 0 0 0 0 0
0 0 0 0 0 0 0 0 0 0 0
T4T139 0 -9.575013 -35.655285 6 126 0 0 0 0 0 0
0 0 0 0 0 0 0 0 0 0 0
T4T156 0 -9.623859 -35.693058 9 241.4 0 0 0 0.037282519
0.064285714 9 0.182270091 0.314285714 44 0.057995029 0.1 14 0.302402651
0.521428571 73 0.57995029 140
T4T158 0 -9.503634 -35.579916 6 93.5 0 0 0 0.053475936
0.131578947 5 0.117647059 0.289473684 11 0.032085561 0.078947368 3

0.203208556 0.5 19 0.406417112 38
T4T161 0 -9.56846 -35.6494 6 126 0 0 0 0 0 0
0 0 0 0 0 0 0 0 0 0 0
T4T163 0 -9.627673 -35.694645 6 160 0 0 0 0.05625 0.064285714
9 0.275 0.314285714 44 0.0875 0.1 14 0.45625 0.521428571 73 0.875 140
T4T169 0 -9.6153 -35.687809 9 241.4 0 0 0 0.037282519 0.064285714
9 0.182270091 0.314285714 44 0.057995029 0.1 14 0.302402651 0.521428571
73 0.57995029 140
T4T170 0 -9.618101 -35.691562 9 241.4 0 0 0 0.037282519
0.064285714 9 0.182270091 0.314285714 44 0.057995029 0.1 14 0.302402651
0.521428571 73 0.57995029 140
T4T171 0 -9.609355 -35.683301 6 149.4 0 0 0 0 0 0
0 0 0 0 0 0 0 0 0 0 0
T4T173 0 -9.535095 -35.610125 6 75.4 0 0 0 0.053050398
0.210526316 4 0.092838196 0.368421053 7 0.026525199 0.105263158 2
0.079575597 0.315789474 6 0.25198939 19
T4T175 0 -9.614233 -35.687061 8 217.2 0 0 0 0.036832413
0.06557377 8 0.174953959 0.31147541 38 0.064456722 0.114754098 14
0.285451197 0.508196721 62 0.561694291 122
T4T179 0 -9.561388 -35.643014 8 151.2 0 0 0 0.026455026 0.25
4 0.046296296 0.4375 7 0.006613757 0.0625 1 0.026455026 0.25 4
0.105820106 16
T4T180 0 -9.615121 -35.687637 9 241.4 0 0 0 0.037282519
0.064285714 9 0.182270091 0.314285714 44 0.057995029 0.1 14 0.302402651
0.521428571 73 0.57995029 140
T4T181 0 -9.618204 -35.68985 9 241.4 0 0 0 0.037282519
0.064285714 9 0.182270091 0.314285714 44 0.057995029 0.1 14 0.302402651
0.521428571 73 0.57995029 140
T4T182 0 -9.5317 -35.605971 4 47.7 0 0 0 0.083857442 0.210526316
4 0.146750524 0.368421053 7 0.041928721 0.105263158 2 0.125786164
0.315789474 6 0.398322851 19
T4T183 0 -9.525472 -35.591433 6 93.5 0 0 0 0.053475936
0.131578947 5 0.117647059 0.289473684 11 0.032085561 0.078947368 3
0.203208556 0.5 19 0.406417112 38
T4T196 0 -9.625994 -35.694024 9 241.4 0 0 0 0.037282519
0.064285714 9 0.182270091 0.314285714 44 0.057995029 0.1 14 0.302402651
0.521428571 73 0.57995029 140
T4T208 0 -9.555167 -35.635058 6 75.4 0 0 0 0.053050398

0.210526316 4 0.092838196 0.368421053 7 0.026525199 0.105263158 2
0.079575597 0.315789474 6 0.25198939 19
T4T211 0 -9.515279 -35.589087 6 93.5 0 0 0 0.053475936
0.131578947 5 0.117647059 0.289473684 11 0.032085561 0.078947368 3
0.203208556 0.5 19 0.406417112 38
T4T215 0 -9.622804 -35.692587 9 241.4 0 0 0 0.037282519
0.064285714 9 0.182270091 0.314285714 44 0.057995029 0.1 14 0.302402651
0.521428571 73 0.57995029 140
T4T219 0 -9.627907 -35.694869 9 241.4 0 0 0 0.037282519
0.064285714 9 0.182270091 0.314285714 44 0.057995029 0.1 14 0.302402651
0.521428571 73 0.57995029 140
T4T222 0 -9.628869 -35.695081 9 241.4 0 0 0 0.037282519
0.064285714 9 0.182270091 0.314285714 44 0.057995029 0.1 14 0.302402651
0.521428571 73 0.57995029 140
T4T225 0 -9.591404 -35.666757 9 194 0 0 0 0 0 0
0 0 0 0 0 0 0 0 0 0 0
T4T229 0 -9.541856 -35.615075 6 75.4 0 0 0 0.053050398
0.210526316 4 0.092838196 0.368421053 7 0.026525199 0.105263158 2
0.079575597 0.315789474 6 0.25198939 19
T4T232 0 -9.537931 -35.613065 6 75.4 0 0 0 0.053050398
0.210526316 4 0.092838196 0.368421053 7 0.026525199 0.105263158 2
0.079575597 0.315789474 6 0.25198939 19
T4T235 0 -9.572663 -35.654822 6 126 0 0 0 0 0 0
0 0 0 0 0 0 0 0 0 0 0
T4T252 0 -9.608428 -35.680373 6 149.4 0 0 0 0 0 0
0 0 0 0 0 0 0 0 0 0 0
T4T254 0 -9.608428 -35.680373 6 149.4 0 0 0 0 0 0
0 0 0 0 0 0 0 0 0 0 0
T4T269 0 -9.520683 -35.591137 6 93.5 0 0 0 0.053475936
0.131578947 5 0.117647059 0.289473684 11 0.032085561 0.078947368 3
0.203208556 0.5 19 0.406417112 38
T4T278 0 -9.530348 -35.602568 6 93.5 0 0 0 0.053475936
0.131578947 5 0.117647059 0.289473684 11 0.032085561 0.078947368 3
0.203208556 0.5 19 0.406417112 38
T4T279 0 -9.619319 -35.690492 9 241.4 0 0 0 0.037282519
0.064285714 9 0.182270091 0.314285714 44 0.057995029 0.1 14 0.302402651
0.521428571 73 0.57995029 140
T4T281 0 -9.574322 -35.655065 6 126 0 0 0 0 0 0

```
0	0	0	0	0	0	0	0	0	0	0
T4T37	0	-9.510886	-35.586717	6	93.5	0	0	0	0.053475936
0.131578947	5	0.117647059	0.289473684	11	0.032085561	0.078947368	3
0.203208556	0.5	19	0.406417112	38
T4T38	0	-9.49143	-35.563283	6	128.2	0.007800312	0.023809524	1
0.03900156	0.119047619	5	0.070202808	0.214285714	9	0.007800312	0.023809524
1	0.202808112	0.619047619	26	0.327613105	42
T4T84	0	-9.531775	-35.606013	5	76	0	0	0	0.052631579	0.16
4	0.105263158	0.32	8	0.026315789	0.08	2	0.144736842	0.44	11
0.328947368	25
T4T98	0	-9.485751	-35.557179	6	128.2	0.007800312	0.023809524	1
0.03900156	0.119047619	5	0.070202808	0.214285714	9	0.007800312	0.023809524
1	0.202808112	0.619047619	26	0.327613105	42
T5T113	0	-9.655446	-35.698203	6	160	0	0	0	0.05625	0.064285714
9	0.275	0.314285714	44	0.0875	0.1	14	0.45625	0.521428571	73	0.875	140
T5T114	0	-9.655529	-35.698219	6	160	0	0	0	0.05625	0.064285714
9	0.275	0.314285714	44	0.0875	0.1	14	0.45625	0.521428571	73	0.875	140
T5T123	0	-9.65668	-35.723784	3	92	0	0	0	0.097826087
0.064285714	9	0.47826087	0.314285714	44	0.152173913	0.1	14	0.793478261
0.521428571	73	1.52173913	140
T5T130	0	-9.663564	-35.695428	4	116.5	0	0	0	0.077253219
0.064285714	9	0.377682403	0.314285714	44	0.120171674	0.1	14	0.626609442
0.521428571	73	1.201716738	140
T5T131	0	-9.655653	-35.698278	6	160	0	0	0	0.05625	0.064285714
9	0.275	0.314285714	44	0.0875	0.1	14	0.45625	0.521428571	73	0.875	140
T5T139	0	-9.659807	-35.697149	6	160	0	0	0	0.05625	0.064285714
9	0.275	0.314285714	44	0.0875	0.1	14	0.45625	0.521428571	73	0.875	140
T5T147	0	-9.647724	-35.699458	6	160	0	0	0	0.05625	0.064285714
9	0.275	0.314285714	44	0.0875	0.1	14	0.45625	0.521428571	73	0.875	140
T5T148	0	-9.639705	-35.698147	6	160	0	0	0	0.05625	0.064285714
9	0.275	0.314285714	44	0.0875	0.1	14	0.45625	0.521428571	73	0.875	140
T5T157	0	-9.676358	-35.718402	3	92	0	0	0	0.097826087
0.064285714	9	0.47826087	0.314285714	44	0.152173913	0.1	14	0.793478261
0.521428571	73	1.52173913	140
T5T164	0	-9.653834	-35.698607	6	160	0	0	0	0.05625	0.064285714
9	0.275	0.314285714	44	0.0875	0.1	14	0.45625	0.521428571	73	0.875	140
T5T177	0	-9.649117	-35.699699	6	160	0	0	0	0.05625	0.064285714
9	0.275	0.314285714	44	0.0875	0.1	14	0.45625	0.521428571	73	0.875	140
```

T5T179 0 -9.651655 -35.699 6 160 0 0 0 0.05625 0.064285714 9
0.275 0.314285714 44 0.0875 0.1 14 0.45625 0.521428571 73 0.875 140
T5T78 0 -9.673577 -35.716616 3 92 0 0 0 0.097826087
0.064285714 9 0.47826087 0.314285714 44 0.152173913 0.1 14 0.793478261
0.521428571 73 1.52173913 140
T5T79 0 -9.644858 -35.69905 6 160 0 0 0 0.05625 0.064285714
9 0.275 0.314285714 44 0.0875 0.1 14 0.45625 0.521428571 73 0.875 140
T5T86 0 -9.671352 -35.715059 3 92 0 0 0 0.097826087
0.064285714 9 0.47826087 0.314285714 44 0.152173913 0.1 14 0.793478261
0.521428571 73 1.52173913 140
T5T88 0 -9.659262 -35.697765 6 160 0 0 0 0.05625 0.064285714
9 0.275 0.314285714 44 0.0875 0.1 14 0.45625 0.521428571 73 0.875 140
T5T91 0 -9.648369 -35.699923 6 160 0 0 0 0.05625 0.064285714
9 0.275 0.314285714 44 0.0875 0.1 14 0.45625 0.521428571 73 0.875 140
T1T13-20 1 -8.991264 -35.199513 14 168.26 0.035659099 0.030612245 6
0.172352312 0.147959184 29 0.416022822 0.357142857 70 0.101034114 0.086734694
17 0.439795554 0.37755102 74 1.164863901 196
T1T39 1 -9.13933 -35.286916 9 221.2 0.004520796 0.007874016 1
0.158227848 0.275590551 35 0.2079566 0.362204724 46 0.099457505 0.173228346
22 0.1039783 0.181102362 23 0.574141049 127
T1T78 1 -9.129348 -35.283352 9 221.2 0.004520796 0.007874016 1
0.158227848 0.275590551 35 0.2079566 0.362204724 46 0.099457505 0.173228346
22 0.1039783 0.181102362 23 0.574141049 127
T2T102 1 -9.245584 -35.348504 3 86.8 0.011520737 0.016949153 1
0.046082949 0.06779661 4 0.184331797 0.271186441 16 0.023041475 0.033898305
2 0.414746544 0.610169492 36 0.679723502 59
T2T11-20 1 -9.25507 -35.354339 3 86.8 0.011520737 0.016949153 1
0.046082949 0.06779661 4 0.184331797 0.271186441 16 0.023041475 0.033898305
2 0.414746544 0.610169492 36 0.679723502 59
T2T17-20 1 -9.189878 -35.30459 6 79.6 0.037688442 0.056603774 3
0.125628141 0.188679245 10 0.125628141 0.188679245 10 0.150753769 0.226415094
12 0.226130653 0.339622642 18 0.665829146 53
T2T5-20 1 -9.21012 -35.3244 6 116.6 0.034305317 0.05 4 0.051457976
0.075 6 0.180102916 0.2625 21 0.025728988 0.0375 3 0.394511149 0.575 46
0.686106346 80
T2T62 1 -9.21699 -35.32817 6 166.6 0.024009604 0.05 4 0.036014406
0.075 6 0.12605042 0.2625 21 0.018007203 0.0375 3 0.276110444 0.575 46
0.480192077 80

T2T86 1 -9.214594 -35.327403 6 116.6 0.034305317 0.05 4 0.051457976
0.075 6 0.180102916 0.2625 21 0.025728988 0.0375 3 0.394511149 0.575 46
0.686106346 80
T2T95 1 -9.289272 -35.388182 6 114.6 0 0 0 0.034904014
0.072727273 4 0.235602094 0.490909091 27 0.052356021 0.109090909 6
0.157068063 0.327272727 18 0.479930192 55
T3T101 1 -9.465226 -35.542781 6 128.2 0.007800312 0.023809524 1
0.03900156 0.119047619 5 0.070202808 0.214285714 9 0.007800312 0.023809524
1 0.202808112 0.619047619 26 0.327613105 42
T3T113 1 -9.390735 -35.495148 3 88 0.011363636 0.011494253 1
0.159090909 0.16091954 14 0.352272727 0.356321839 31 0.136363636 0.137931034
12 0.329545455 0.333333333 29 0.988636364 87
T3T123 1 -9.408554 -35.496621 3 88 0.011363636 0.011494253 1
0.159090909 0.16091954 14 0.352272727 0.356321839 31 0.136363636 0.137931034
12 0.329545455 0.333333333 29 0.988636364 87
T3T124 1 -9.440253 -35.516452 6 153.5 0.013029316 0.018181818 2
0.117263844 0.163636364 18 0.234527687 0.327272727 36 0.078175896 0.109090909
12 0.273615635 0.381818182 42 0.716612378 110
T3T47 1 -9.45149 -35.529575 3 65.5 0.015267176 0.043478261 1
0.061068702 0.173913043 4 0.076335878 0.217391304 5 0 0 0
0.198473282 0.565217391 13 0.351145038 23
T3T82 1 -9.420504 -35.503515 3 88 0.011363636 0.011494253 1
0.159090909 0.16091954 14 0.352272727 0.356321839 31 0.136363636 0.137931034
12 0.329545455 0.333333333 29 0.988636364 87
T4T130 1 -9.59221 -35.6679 9 194 0 0 0 0 0 0
0 0 0 0 0 0 0 0 0 0 0
T4T136 1 -9.598346 -35.675225 6 149.4 0 0 0 0 0 0
0 0 0 0 0 0 0 0 0 0 0
T4T160 1 -9.55033 -35.63046 6 75.4 0 0 0 0.053050398
0.210526316 4 0.092838196 0.368421053 7 0.026525199 0.105263158 2
0.079575597 0.315789474 6 0.25198939 19
T4T184 1 -9.62329 -35.692995 9 241.4 0 0 0 0.037282519
0.064285714 9 0.182270091 0.314285714 44 0.057995029 0.1 14 0.302402651
0.521428571 73 0.57995029 140
T4T245 1 -9.488383 -35.559275 6 128.2 0.007800312 0.023809524 1
0.03900156 0.119047619 5 0.070202808 0.214285714 9 0.007800312 0.023809524
1 0.202808112 0.619047619 26 0.327613105 42
T4T251 1 -9.575229 -35.655252 5 108.2 0 0 0 0 0 0

0	0	0	0	0	0	0	0	0	0	0
T4T255	1	-9.634273	-35.697166	6	149.4	0	0	0	0	0	0
0	0	0	0	0	0	0	0	0	0	0
T4T266	1	-9.588247	-35.664661	9	194	0	0	0	0	0	0
0	0	0	0	0	0	0	0	0	0	0
T4T284	1	-9.63733	-35.697983	6	160	0	0	0	0.05625	0.064285714
9	0.275	0.314285714	44	0.0875	0.1	14	0.45625	0.521428571	73	0.875	140
T4T58	1	-9.602283	-35.67808	9	241.4	0	0	0	0.037282519
0.064285714	9	0.182270091	0.314285714	44	0.057995029	0.1	14	0.302402651
0.521428571	73	0.57995029	140
T4T99	1	-9.619676	-35.691062	9	241.4	0	0	0	0.037282519
0.064285714	9	0.182270091	0.314285714	44	0.057995029	0.1	14	0.302402651
0.521428571	73	0.57995029	140
T5T115	1	-9.668196	-35.71234	9	241.4	0	0	0	0.037282519
0.064285714	9	0.182270091	0.314285714	44	0.057995029	0.1	14	0.302402651
0.521428571	73	0.57995029	140
T5T120	1	-9.664972	-35.709048	3	92	0	0	0	0.097826087
0.064285714	9	0.47826087	0.314285714	44	0.152173913	0.1	14	0.793478261
0.521428571	73	1.52173913	140
T5T149	1	-9.648201	-35.699974	6	160	0	0	0	0.05625	0.064285714
9	0.275	0.314285714	44	0.0875	0.1	14	0.45625	0.521428571	73	0.875	140
T5T80	1	-9.655629	-35.69823	6	160	0	0	0	0.05625	0.064285714
9	0.275	0.314285714	44	0.0875	0.1	14	0.45625	0.521428571	73	0.875	140
T5T9	1	-9.6731	-35.7158	3	92	0	0	0	0.097826087	0.064285714
9	0.47826087	0.314285714	44	0.152173913	0.1	14	0.793478261	0.521428571
73	1.52173913	140

DATASET: plastic_transects_number

Turtle_ID	prob	Lat	Long	N_transects	Total_Transect_area_m2	dens_nylon	porc_nylon	transect_Nylon/rope	dens_styrofoam	porc_styrofoam	transect_Styrofoam	dens_flex	porc_flex	transect_Flex	dens_hard	porc_hard	transect_Hard	dens_other	porc_other	transect_Others	total_density	transect_Total_items	Total_items
T1T107	0	-9.140839	-35.287237	9	221.2	0.004520796	0.007874016	1	0.158227848	0.275590551	35	0.2079566	0.362204724	46	0.099457505	0.173228346	22	0.1039783	0.181102362	23	0.574141049	127	0
T1T110	0	-8.944311	-35.169845	15	215.66	0.027821571	0.034482759	6	0.180840211	0.224137931	39	0.324584995	0.402298851	70	0.06028007	0.074712644	13	0.213298711	0.264367816	46	0.806825559	174	0
T1T116	0	-9.045179	-35.236564	8	179.6	0.022271715	0.022857143	4	0.13363029	0.137142857	24	0.267260579	0.274285714	48	0.139198218	0.142857143	25	0.412026726	0.422857143	74	0.974387528	175	0
T1T117	0	-9.045179	-35.236564	8	179.6	0.022271715	0.022857143	4	0.13363029	0.137142857	24	0.267260579	0.274285714	48	0.139198218	0.142857143	25	0.412026726	0.422857143	74	0.974387528	175	0
T1T119	0	-9.132235	-35.285097	6	129.8	0.00770416	0.015151515	1	0.154083205	0.303030303	20	0.092449923	0.181818182	12	0.138674884	0.272727273	18	0.115562404	0.227272727	15	0.508474576	66	0
T1T121	0	-9.083247	-35.250767	9	250.6	0.023942538	0.034090909	6	0.127693536	0.181818182	32	0.159616919	0.227272727	40	0.11971269	0.170454545	30	0.271348763	0.386363636	68	0.702314445	176	0
T1T122	0	-8.914256	-35.152264	7	128.1	0.023419204	0.035714286	3	0.148321624	0.226190476	19	0.218579235	0.333333333	28	0.06245121	0.095238095	8	0.202966432	0.30952381	26	0.655737705	84	0
T1T123	0	-9.004481	-35.214375	9	118.06	0.033881077	0.024539877	4	0.152464848	0.110429448	18	0.5251567	0.380368098	62	0.143994579	0.104294479	17	0.5251567	0.380368098	62	1.380653905	163	0
T1T48	0	-8.963819	-35.175211	15	200.06	0.034989503	0.044585987	7	0.139958013	0.178343949	28	0.32990103	0.420382166	66	0.044986504	0.057324841	9	0.234929521	0.299363057	47	0.784764571	157	1
T1T7-20	0	-8.928337	-35.162035	12	178.3	0.028042625	0.042735043	5	0.168255749	0.256410256	30	0.201906898	0.307692308	36	0.0448682	0.068376068	8	0.213123948	0.324786325	38	0.65619742	117	1
T1T76	0	-9.129348	-35.283353	9	221.2	0.004520796	0.007874016	1	0.158227848	0.275590551	35	0.2079566	0.362204724	46	0.099457505	0.173228346	22	0.1039783	0.181102362	23	0.574141049	127	0
T1T79	0	-9.129348	-35.283352	9	221.2	0.004520796	0.007874016	1															

0.158227848	0.275590551	35	0.2079566	0.362204724	46	0.099457505	0.173228346
22	0.1039783	0.181102362	23	0.574141049	127	0
T1T85	0	-8.999937	-35.208354	9	118.06	0.033881077	0.024539877	4
0.152464848	0.110429448	18	0.5251567	0.380368098	62	0.143994579	0.104294479
17	0.5251567	0.380368098	62	1.380653905	163	0
T1T91	0	-9.054997	-35.237124	6	170.6	0.029308324	0.035211268	5
0.117233294	0.14084507	20	0.193434936	0.232394366	33	0.134818288	0.161971831
23	0.357561547	0.429577465	61	0.832356389	142	2
T1T9-20	0	-8.991589	-35.199852	14	168.28	0.035654861	0.030612245	6
0.172331828	0.147959184	29	0.415973378	0.357142857	70	0.101022106	0.086734694
17	0.439743285	0.37755102	74	1.164725458	196	0
T1T98	0	-9.045149	-35.236929	8	179.6	0.033407572	0.028571429	6
0.122494432	0.104761905	22	0.278396437	0.238095238	50	0.183741648	0.157142857
33	0.551224944	0.471428571	99	1.169265033	210	0
T2T100	0	-9.224858	-35.332822	6	116.6	0.034305317	0.05	4	0.051457976
0.075	6	0.180102916	0.2625	21	0.025728988	0.0375	3	0.394511149	0.575	46
0.686106346	80	0
T2T103	0	-9.208261	-35.325023	6	116.6	0.034305317	0.05	4	0.051457976
0.075	6	0.180102916	0.2625	21	0.025728988	0.0375	3	0.394511149	0.575	46
0.686106346	80	0
T2T104	0	-9.179131	-35.298745	6	79.6	0.037688442	0.056603774	3
0.125628141	0.188679245	10	0.125628141	0.188679245	10	0.150753769	0.226415094
12	0.226130653	0.339622642	18	0.665829146	53	0
T2T105	0	-9.229095	-35.340251	6	116.6	0.034305317	0.05	4	0.051457976
0.075	6	0.180102916	0.2625	21	0.025728988	0.0375	3	0.394511149	0.575	46
0.686106346	80	0
T2T112	0	-9.270559	-35.367935	5	109.4	0.036563071	0.050632911	4
0.054844607	0.075949367	6	0.191956124	0.265822785	21	0.027422303	0.037974684
3	0.411334552	0.569620253	45	0.722120658	79	1
T2T1-20	0	-9.26485	-35.36348	6	139	0.007194245	0.0125	1	0.043165468
0.075	6	0.172661871	0.3	24	0.014388489	0.025	2	0.338129496	0.5875	47
0.575539568	80	0
T2T121	0	-9.200526	-35.320901	3	29.8	0.100671141	0.142857143	3
0.067114094	0.095238095	2	0.167785235	0.238095238	5	0.033557047	0.047619048
1	0.33557047	0.476190476	10	0.704697987	21	0
T2T123	0	-9.199497	-35.320291	3	29.8	0.100671141	0.142857143	3
0.067114094	0.095238095	2	0.167785235	0.238095238	5	0.033557047	0.047619048
1	0.33557047	0.476190476	10	0.704697987	21	0

T2T16-20 0 -9.119172 -35.269427 12 326 0.009202454 0.014851485 3
0.153374233 0.247524752 50 0.17791411 0.287128713 58 0.104294479 0.168316832
34 0.174846626 0.282178218 57 0.619631902 202 0
T2T18-20 0 -9.279354 -35.376816 6 139 0.007194245 0.0125 1
0.043165468 0.075 6 0.172661871 0.3 24 0.014388489 0.025 2 0.338129496
0.5875 47 0.575539568 80 1
T2T2-20 0 -9.20967 -35.32439 6 116.6 0.034305317 0.05 4 0.051457976
0.075 6 0.180102916 0.2625 21 0.025728988 0.0375 3 0.394511149 0.575 46
0.686106346 80 0
T2T26 0 -9.215828 -35.327898 6 116.6 0.034305317 0.05 4 0.051457976
0.075 6 0.180102916 0.2625 21 0.025728988 0.0375 3 0.394511149 0.575 46
0.686106346 80 0
T2T3-20 0 -9.20646 -35.32382 5 58.2 0.068728522 0.083333333 4
0.068728522 0.083333333 4 0.223367698 0.270833333 13 0.034364261 0.041666667
2 0.429553265 0.520833333 25 0.824742268 48 0
T2T33 0 -9.242848 -35.346781 3 86.8 0.011520737 0.016949153 1
0.046082949 0.06779661 4 0.184331797 0.271186441 16 0.023041475 0.033898305
2 0.414746544 0.610169492 36 0.679723502 59 0
T2T37 0 -9.23957 -35.34407 3 86.8 0.011520737 0.016949153 1
0.046082949 0.06779661 4 0.184331797 0.271186441 16 0.023041475 0.033898305
2 0.414746544 0.610169492 36 0.679723502 59 1
T2T38 0 -9.18535 -35.301591 6 79.6 0.037688442 0.056603774 3
0.125628141 0.188679245 10 0.125628141 0.188679245 10 0.150753769 0.226415094
12 0.226130653 0.339622642 18 0.665829146 53 0
T2T41 0 -9.221212 -35.341857 6 116.6 0.034305317 0.05 4 0.051457976
0.075 6 0.180102916 0.2625 21 0.025728988 0.0375 3 0.394511149 0.575 46
0.686106346 80 0
T2T49 0 -9.19589 -35.312568 3 29.8 0.100671141 0.142857143 3
0.067114094 0.095238095 2 0.167785235 0.238095238 5 0.033557047 0.047619048
1 0.33557047 0.476190476 10 0.704697987 21 0
T2T55 0 -9.237506 -35.343066 3 86.8 0.011520737 0.016949153 1
0.046082949 0.06779661 4 0.184331797 0.271186441 16 0.023041475 0.033898305
2 0.414746544 0.610169492 36 0.679723502 59 0
T2T6-20 0 -9.23426 -35.34106 3 86.8 0.011520737 0.016949153 1
0.046082949 0.06779661 4 0.184331797 0.271186441 16 0.023041475 0.033898305
2 0.414746544 0.610169492 36 0.679723502 59 0
T2T63 0 -9.216961 -35.328175 6 166.6 0.024009604 0.05 4 0.036014406
0.075 6 0.12605042 0.2625 21 0.018007203 0.0375 3 0.276110444 0.575 46

0.480192077 80 0
T2T7-20 0 -9.23545 -35.34208 3 86.8 0.011520737 0.016949153 1
0.046082949 0.06779661 4 0.184331797 0.271186441 16 0.023041475 0.033898305
2 0.414746544 0.610169492 36 0.679723502 59 0
T2T81 0 -9.188453 -335.303289 6 79.6 0.037688442 0.056603774 3
0.125628141 0.188679245 10 0.125628141 0.188679245 10 0.150753769 0.226415094
12 0.226130653 0.339622642 18 0.665829146 53 0
T2T8-20 0 -9.24274 -35.34645 3 86.8 0.011520737 0.016949153 1
0.046082949 0.06779661 4 0.184331797 0.271186441 16 0.023041475 0.033898305
2 0.414746544 0.610169492 36 0.679723502 59 0
T2T85 0 -9.261948 -35.360342 4 102.2 0.009784736 0.016393443 1
0.039138943 0.06557377 4 0.176125245 0.295081967 18 0.019569472 0.032786885
2 0.352250489 0.590163934 36 0.596868885 61 1
T2T88 0 -9.175941 -35.297863 7 109.2 0.027472527 0.044776119 3
0.10989011 0.179104478 12 0.192307692 0.313432836 21 0.119047619 0.194029851
13 0.164835165 0.268656716 18 0.613553114 67 0
T2T9-20 0 -9.27877 -35.37619 6 139 0.007194245 0.0125 1 0.043165468
0.075 6 0.172661871 0.3 24 0.014388489 0.025 2 0.338129496 0.5875 47
0.575539568 80 1
T2T96 0 -9.219314 -35.328873 6 166.6 0.024009604 0.05 4 0.036014406
0.075 6 0.12605042 0.2625 21 0.018007203 0.0375 3 0.276110444 0.575 46
0.480192077 80 0
T2T97 0 -9.264018 -35.362634 6 139 0.007194245 0.0125 1 0.043165468
0.075 6 0.172661871 0.3 24 0.014388489 0.025 2 0.338129496 0.5875 47
0.575539568 80 0
T3T108 0 -9.450684 -35.529718 3 65.5 0.015267176 0.043478261 1
0.061068702 0.173913043 4 0.076335878 0.217391304 5 0 0 0
0.198473282 0.565217391 13 0.351145038 23 0
T3T109 0 -9.428528 -35.5064 3 88 0.011363636 0.011494253 1
0.159090909 0.16091954 14 0.352272727 0.356321839 31 0.136363636 0.137931034
12 0.329545455 0.333333333 29 0.988636364 87 0
T3T117 0 -9.450042 -35.529725 3 65.5 0.015267176 0.043478261 1
0.061068702 0.173913043 4 0.076335878 0.217391304 5 0 0 0
0.198473282 0.565217391 13 0.351145038 23 0
T3T12 0 -9.474221 -35.553936 6 128.2 0.007800312 0.023809524 1
0.03900156 0.119047619 5 0.070202808 0.214285714 9 0.007800312 0.023809524
1 0.202808112 0.619047619 26 0.327613105 42 0
T3T121 0 -9.474288 -35.553246 6 128.2 0.007800312 0.023809524 1

0.03900156 0.119047619 5 0.070202808 0.214285714 9 0.007800312 0.023809524
1 0.202808112 0.619047619 26 0.327613105 42 2
T3T125 0 -9.465833333 -35.54333333 6 128.2 0.007800312 0.023809524 1
0.03900156 0.119047619 5 0.070202808 0.214285714 9 0.007800312 0.023809524
1 0.202808112 0.619047619 26 0.327613105 42 0
T3T37 0 -9.431846 -35.509543 4 113.6 0.017605634 0.021505376 2
0.132042254 0.161290323 15 0.281690141 0.344086022 32 0.105633803 0.129032258
12 0.281690141 0.344086022 32 0.818661972 93 0
T3T50 0 -9.400441 -35.496373 3 88 0.011363636 0.011494253 1
0.159090909 0.16091954 14 0.352272727 0.356321839 31 0.136363636 0.137931034
12 0.329545455 0.333333333 29 0.988636364 87 1
T3T65 0 -9.400327 -35.496336 3 88 0.011363636 0.011494253 1
0.159090909 0.16091954 14 0.352272727 0.356321839 31 0.136363636 0.137931034
12 0.329545455 0.333333333 29 0.988636364 87 0
T3T73 0 -9.411194 -35.506696 3 88 0.011363636 0.011494253 1
0.159090909 0.16091954 14 0.352272727 0.356321839 31 0.136363636 0.137931034
12 0.329545455 0.333333333 29 0.988636364 87 0
T3T79 0 -9.411206 -35.506659 3 88 0.011363636 0.011494253 1
0.159090909 0.16091954 14 0.352272727 0.356321839 31 0.136363636 0.137931034
12 0.329545455 0.333333333 29 0.988636364 87 1
T4T112 0 -9.541113 -35.61442 6 75.4 0 0 0 0.053050398
0.210526316 4 0.092838196 0.368421053 7 0.026525199 0.105263158 2
0.079575597 0.315789474 6 0.25198939 19 0
T4T124 0 -9.620266 -35.691299 9 241.4 0 0 0 0.037282519
0.064285714 9 0.182270091 0.314285714 44 0.057995029 0.1 14 0.302402651
0.521428571 73 0.57995029 140 0
T4T131 0 -9.61185 -35.68501 8 217.2 0 0 0 0.036832413
0.06557377 8 0.174953959 0.31147541 38 0.064456722 0.114754098 14
0.285451197 0.508196721 62 0.561694291 122 1
T4T137 0 -9.575048 -35.655268 6 126 0 0 0 0 0 0
0 0 0 0 0 0 0 0 0 0 0 0
T4T139 0 -9.575013 -35.655285 6 126 0 0 0 0 0 0
0 0 0 0 0 0 0 0 0 0 0 0
T4T156 0 -9.623859 -35.693058 9 241.4 0 0 0 0.037282519
0.064285714 9 0.182270091 0.314285714 44 0.057995029 0.1 14 0.302402651
0.521428571 73 0.57995029 140 0
T4T158 0 -9.503634 -35.579916 6 93.5 0 0 0 0.053475936
0.131578947 5 0.117647059 0.289473684 11 0.032085561 0.078947368 3

0.203208556 0.5 19 0.406417112 38 1
T4T161 0 -9.56846 -35.6494 6 126 0 0 0 0 0 0
0 0 0 0 0 0 0 0 0 0 0 2
T4T163 0 -9.627673 -35.694645 6 160 0 0 0 0.05625 0.064285714
9 0.275 0.314285714 44 0.0875 0.1 14 0.45625 0.521428571 73 0.875 140
0
T4T169 0 -9.6153 -35.687809 9 241.4 0 0 0 0.037282519 0.064285714
9 0.182270091 0.314285714 44 0.057995029 0.1 14 0.302402651 0.521428571
73 0.57995029 140 0
T4T170 0 -9.618101 -35.691562 9 241.4 0 0 0 0.037282519
0.064285714 9 0.182270091 0.314285714 44 0.057995029 0.1 14 0.302402651
0.521428571 73 0.57995029 140 2
T4T171 0 -9.609355 -35.683301 6 149.4 0 0 0 0 0 0
0 0 0 0 0 0 0 0 0 0 0 0
T4T173 0 -9.535095 -35.610125 6 75.4 0 0 0 0.053050398
0.210526316 4 0.092838196 0.368421053 7 0.026525199 0.105263158 2
0.079575597 0.315789474 6 0.25198939 19 0
T4T175 0 -9.614233 -35.687061 8 217.2 0 0 0 0.036832413
0.06557377 8 0.174953959 0.31147541 38 0.064456722 0.114754098 14
0.285451197 0.508196721 62 0.561694291 122 0
T4T179 0 -9.561388 -35.643014 8 151.2 0 0 0 0.026455026 0.25
4 0.046296296 0.4375 7 0.006613757 0.0625 1 0.026455026 0.25 4
0.105820106 16 0
T4T180 0 -9.615121 -35.687637 9 241.4 0 0 0 0.037282519
0.064285714 9 0.182270091 0.314285714 44 0.057995029 0.1 14 0.302402651
0.521428571 73 0.57995029 140 1
T4T181 0 -9.618204 -35.68985 9 241.4 0 0 0 0.037282519
0.064285714 9 0.182270091 0.314285714 44 0.057995029 0.1 14 0.302402651
0.521428571 73 0.57995029 140 0
T4T182 0 -9.5317 -35.605971 4 47.7 0 0 0 0.083857442 0.210526316
4 0.146750524 0.368421053 7 0.041928721 0.105263158 2 0.125786164
0.315789474 6 0.398322851 19 0
T4T183 0 -9.525472 -35.591433 6 93.5 0 0 0 0.053475936
0.131578947 5 0.117647059 0.289473684 11 0.032085561 0.078947368 3
0.203208556 0.5 19 0.406417112 38 1
T4T196 0 -9.625994 -35.694024 9 241.4 0 0 0 0.037282519
0.064285714 9 0.182270091 0.314285714 44 0.057995029 0.1 14 0.302402651
0.521428571 73 0.57995029 140 0

T4T208 0 -9.555167 -35.635058 6 75.4 0 0 0 0.053050398
0.210526316 4 0.092838196 0.368421053 7 0.026525199 0.105263158 2
0.079575597 0.315789474 6 0.25198939 19 1
T4T211 0 -9.515279 -35.589087 6 93.5 0 0 0 0.053475936
0.131578947 5 0.117647059 0.289473684 11 0.032085561 0.078947368 3
0.203208556 0.5 19 0.406417112 38 0
T4T215 0 -9.622804 -35.692587 9 241.4 0 0 0 0.037282519
0.064285714 9 0.182270091 0.314285714 44 0.057995029 0.1 14 0.302402651
0.521428571 73 0.57995029 140 0
T4T219 0 -9.627907 -35.694869 9 241.4 0 0 0 0.037282519
0.064285714 9 0.182270091 0.314285714 44 0.057995029 0.1 14 0.302402651
0.521428571 73 0.57995029 140 0
T4T222 0 -9.628869 -35.695081 9 241.4 0 0 0 0.037282519
0.064285714 9 0.182270091 0.314285714 44 0.057995029 0.1 14 0.302402651
0.521428571 73 0.57995029 140 0
T4T225 0 -9.591404 -35.666757 9 194 0 0 0 0 0 0
0 0 0 0 0 0 0 0 0 0 0 2
T4T229 0 -9.541856 -35.615075 6 75.4 0 0 0 0.053050398
0.210526316 4 0.092838196 0.368421053 7 0.026525199 0.105263158 2
0.079575597 0.315789474 6 0.25198939 19 0
T4T232 0 -9.537931 -35.613065 6 75.4 0 0 0 0.053050398
0.210526316 4 0.092838196 0.368421053 7 0.026525199 0.105263158 2
0.079575597 0.315789474 6 0.25198939 19 0
T4T235 0 -9.572663 -35.654822 6 126 0 0 0 0 0 0
0 0 0 0 0 0 0 0 0 0 0 0
T4T252 0 -9.608428 -35.680373 6 149.4 0 0 0 0 0 0
0 0 0 0 0 0 0 0 0 0 0 0
T4T254 0 -9.608428 -35.680373 6 149.4 0 0 0 0 0 0
0 0 0 0 0 0 0 0 0 0 0 0
T4T269 0 -9.520683 -35.591137 6 93.5 0 0 0 0.053475936
0.131578947 5 0.117647059 0.289473684 11 0.032085561 0.078947368 3
0.203208556 0.5 19 0.406417112 38 0
T4T278 0 -9.530348 -35.602568 6 93.5 0 0 0 0.053475936
0.131578947 5 0.117647059 0.289473684 11 0.032085561 0.078947368 3
0.203208556 0.5 19 0.406417112 38 0
T4T279 0 -9.619319 -35.690492 9 241.4 0 0 0 0.037282519
0.064285714 9 0.182270091 0.314285714 44 0.057995029 0.1 14 0.302402651
0.521428571 73 0.57995029 140 0

T4T281 0 -9.574322 -35.655065 6 126 0 0 0 0 0 0
0 0 0 0 0 0 0 0 0 0 0 0
T4T37 0 -9.510886 -35.586717 6 93.5 0 0 0 0.053475936
0.131578947 5 0.117647059 0.289473684 11 0.032085561 0.078947368 3
0.203208556 0.5 19 0.406417112 38 0
T4T38 0 -9.49143 -35.563283 6 128.2 0.007800312 0.023809524 1
0.03900156 0.119047619 5 0.070202808 0.214285714 9 0.007800312 0.023809524
1 0.202808112 0.619047619 26 0.327613105 42 0
T4T84 0 -9.531775 -35.606013 5 76 0 0 0 0.052631579 0.16
4 0.105263158 0.32 8 0.026315789 0.08 2 0.144736842 0.44 11
0.328947368 25 0
T4T98 0 -9.485751 -35.557179 6 128.2 0.007800312 0.023809524 1
0.03900156 0.119047619 5 0.070202808 0.214285714 9 0.007800312 0.023809524
1 0.202808112 0.619047619 26 0.327613105 42 2
T5T113 0 -9.655446 -35.698203 6 160 0 0 0 0.05625 0.064285714
9 0.275 0.314285714 44 0.0875 0.1 14 0.45625 0.521428571 73 0.875 140
0
T5T114 0 -9.655529 -35.698219 6 160 0 0 0 0.05625 0.064285714
9 0.275 0.314285714 44 0.0875 0.1 14 0.45625 0.521428571 73 0.875 140
0
T5T123 0 -9.65668 -35.723784 3 92 0 0 0 0.097826087
0.064285714 9 0.47826087 0.314285714 44 0.152173913 0.1 14 0.793478261
0.521428571 73 1.52173913 140 0
T5T130 0 -9.663564 -35.695428 4 116.5 0 0 0 0.077253219
0.064285714 9 0.377682403 0.314285714 44 0.120171674 0.1 14 0.626609442
0.521428571 73 1.201716738 140 0
T5T131 0 -9.655653 -35.698278 6 160 0 0 0 0.05625 0.064285714
9 0.275 0.314285714 44 0.0875 0.1 14 0.45625 0.521428571 73 0.875 140
0
T5T139 0 -9.659807 -35.697149 6 160 0 0 0 0.05625 0.064285714
9 0.275 0.314285714 44 0.0875 0.1 14 0.45625 0.521428571 73 0.875 140
0
T5T147 0 -9.647724 -35.699458 6 160 0 0 0 0.05625 0.064285714
9 0.275 0.314285714 44 0.0875 0.1 14 0.45625 0.521428571 73 0.875 140
0
T5T148 0 -9.639705 -35.698147 6 160 0 0 0 0.05625 0.064285714
9 0.275 0.314285714 44 0.0875 0.1 14 0.45625 0.521428571 73 0.875 140
0

T5T157 0 -9.676358 -35.718402 3 92 0 0 0 0.097826087
0.064285714 9 0.47826087 0.314285714 44 0.152173913 0.1 14 0.793478261
0.521428571 73 1.52173913 140 0
T5T164 0 -9.653834 -35.698607 6 160 0 0 0 0.05625 0.064285714
9 0.275 0.314285714 44 0.0875 0.1 14 0.45625 0.521428571 73 0.875 140
0
T5T177 0 -9.649117 -35.699699 6 160 0 0 0 0.05625 0.064285714
9 0.275 0.314285714 44 0.0875 0.1 14 0.45625 0.521428571 73 0.875 140
1
T5T179 0 -9.651655 -35.699 6 160 0 0 0 0.05625 0.064285714 9
0.275 0.314285714 44 0.0875 0.1 14 0.45625 0.521428571 73 0.875 140 5
T5T78 0 -9.673577 -35.716616 3 92 0 0 0 0.097826087
0.064285714 9 0.47826087 0.314285714 44 0.152173913 0.1 14 0.793478261
0.521428571 73 1.52173913 140 0
T5T79 0 -9.644858 -35.69905 6 160 0 0 0 0.05625 0.064285714
9 0.275 0.314285714 44 0.0875 0.1 14 0.45625 0.521428571 73 0.875 140
0
T5T86 0 -9.671352 -35.715059 3 92 0 0 0 0.097826087
0.064285714 9 0.47826087 0.314285714 44 0.152173913 0.1 14 0.793478261
0.521428571 73 1.52173913 140 5
T5T88 0 -9.659262 -35.697765 6 160 0 0 0 0.05625 0.064285714
9 0.275 0.314285714 44 0.0875 0.1 14 0.45625 0.521428571 73 0.875 140
1
T5T91 0 -9.648369 -35.699923 6 160 0 0 0 0.05625 0.064285714
9 0.275 0.314285714 44 0.0875 0.1 14 0.45625 0.521428571 73 0.875 140
0
T1T13-20 1 -8.991264 -35.199513 14 168.26 0.035659099 0.030612245 6
0.172352312 0.147959184 29 0.416022822 0.357142857 70 0.101034114 0.086734694
17 0.439795554 0.37755102 74 1.164863901 196 0
T1T39 1 -9.13933 -35.286916 9 221.2 0.004520796 0.007874016 1
0.158227848 0.275590551 35 0.2079566 0.362204724 46 0.099457505 0.173228346
22 0.1039783 0.181102362 23 0.574141049 127 0
T1T78 1 -9.129348 -35.283352 9 221.2 0.004520796 0.007874016 1
0.158227848 0.275590551 35 0.2079566 0.362204724 46 0.099457505 0.173228346
22 0.1039783 0.181102362 23 0.574141049 127 0
T2T102 1 -9.245584 -35.348504 3 86.8 0.011520737 0.016949153 1
0.046082949 0.06779661 4 0.184331797 0.271186441 16 0.023041475 0.033898305
2 0.414746544 0.610169492 36 0.679723502 59 1

T2T11-20 1 -9.25507 -35.354339 3 86.8 0.011520737 0.016949153 1
0.046082949 0.06779661 4 0.184331797 0.271186441 16 0.023041475 0.033898305
2 0.414746544 0.610169492 36 0.679723502 59 0
T2T17-20 1 -9.189878 -35.30459 6 79.6 0.037688442 0.056603774 3
0.125628141 0.188679245 10 0.125628141 0.188679245 10 0.150753769 0.226415094
12 0.226130653 0.339622642 18 0.665829146 53 0
T2T5-20 1 -9.21012 -35.3244 6 116.6 0.034305317 0.05 4 0.051457976
0.075 6 0.180102916 0.2625 21 0.025728988 0.0375 3 0.394511149 0.575 46
0.686106346 80 3
T2T62 1 -9.21699 -35.32817 6 166.6 0.024009604 0.05 4 0.036014406
0.075 6 0.12605042 0.2625 21 0.018007203 0.0375 3 0.276110444 0.575 46
0.480192077 80 0
T2T86 1 -9.214594 -35.327403 6 116.6 0.034305317 0.05 4 0.051457976
0.075 6 0.180102916 0.2625 21 0.025728988 0.0375 3 0.394511149 0.575 46
0.686106346 80 0
T2T95 1 -9.289272 -35.388182 6 114.6 0 0 0 0.034904014
0.072727273 4 0.235602094 0.490909091 27 0.052356021 0.109090909 6
0.157068063 0.327272727 18 0.479930192 55 13
T3T101 1 -9.465226 -35.542781 6 128.2 0.007800312 0.023809524 1
0.03900156 0.119047619 5 0.070202808 0.214285714 9 0.007800312 0.023809524
1 0.202808112 0.619047619 26 0.327613105 42 0
T3T113 1 -9.390735 -35.495148 3 88 0.011363636 0.011494253 1
0.159090909 0.16091954 14 0.352272727 0.356321839 31 0.136363636 0.137931034
12 0.329545455 0.333333333 29 0.988636364 87 0
T3T123 1 -9.408554 -35.496621 3 88 0.011363636 0.011494253 1
0.159090909 0.16091954 14 0.352272727 0.356321839 31 0.136363636 0.137931034
12 0.329545455 0.333333333 29 0.988636364 87 2
T3T124 1 -9.440253 -35.516452 6 153.5 0.013029316 0.018181818 2
0.117263844 0.163636364 18 0.234527687 0.327272727 36 0.078175896 0.109090909
12 0.273615635 0.381818182 42 0.716612378 110 1
T3T47 1 -9.45149 -35.529575 3 65.5 0.015267176 0.043478261 1
0.061068702 0.173913043 4 0.076335878 0.217391304 5 0 0 0
0.198473282 0.565217391 13 0.351145038 23 0
T3T82 1 -9.420504 -35.503515 3 88 0.011363636 0.011494253 1
0.159090909 0.16091954 14 0.352272727 0.356321839 31 0.136363636 0.137931034
12 0.329545455 0.333333333 29 0.988636364 87 0
T4T130 1 -9.59221 -35.6679 9 194 0 0 0 0 0 0
0 0 0 0 0 0 0 0 0 0 0 0

T4T136 1 -9.598346 -35.675225 6 149.4 0 0 0 0 0 0
0 0 0 0 0 0 0 0 0 0 0 0
T4T160 1 -9.55033 -35.63046 6 75.4 0 0 0 0.053050398
0.210526316 4 0.092838196 0.368421053 7 0.026525199 0.105263158 2
0.079575597 0.315789474 6 0.25198939 19 0
T4T184 1 -9.62329 -35.692995 9 241.4 0 0 0 0.037282519
0.064285714 9 0.182270091 0.314285714 44 0.057995029 0.1 14 0.302402651
0.521428571 73 0.57995029 140 0
T4T245 1 -9.488383 -35.559275 6 128.2 0.007800312 0.023809524 1
0.03900156 0.119047619 5 0.070202808 0.214285714 9 0.007800312 0.023809524
1 0.202808112 0.619047619 26 0.327613105 42 2
T4T251 1 -9.575229 -35.655252 5 108.2 0 0 0 0 0 0
0 0 0 0 0 0 0 0 0 0 0 0
T4T255 1 -9.634273 -35.697166 6 149.4 0 0 0 0 0 0
0 0 0 0 0 0 0 0 0 0 0 0
T4T266 1 -9.588247 -35.664661 9 194 0 0 0 0 0 0
0 0 0 0 0 0 0 0 0 0 0 0
T4T284 1 -9.63733 -35.697983 6 160 0 0 0 0.05625 0.064285714
9 0.275 0.314285714 44 0.0875 0.1 14 0.45625 0.521428571 73 0.875 140
0
T4T58 1 -9.602283 -35.67808 9 241.4 0 0 0 0.037282519
0.064285714 9 0.182270091 0.314285714 44 0.057995029 0.1 14 0.302402651
0.521428571 73 0.57995029 140 0
T4T99 1 -9.619676 -35.691062 9 241.4 0 0 0 0.037282519
0.064285714 9 0.182270091 0.314285714 44 0.057995029 0.1 14 0.302402651
0.521428571 73 0.57995029 140 0
T5T115 1 -9.668196 -35.71234 9 241.4 0 0 0 0.037282519
0.064285714 9 0.182270091 0.314285714 44 0.057995029 0.1 14 0.302402651
0.521428571 73 0.57995029 140 1
T5T120 1 -9.664972 -35.709048 3 92 0 0 0 0.097826087
0.064285714 9 0.47826087 0.314285714 44 0.152173913 0.1 14 0.793478261
0.521428571 73 1.52173913 140 0
T5T149 1 -9.648201 -35.699974 6 160 0 0 0 0.05625 0.064285714
9 0.275 0.314285714 44 0.0875 0.1 14 0.45625 0.521428571 73 0.875 140
0
T5T80 1 -9.655629 -35.69823 6 160 0 0 0 0.05625 0.064285714
9 0.275 0.314285714 44 0.0875 0.1 14 0.45625 0.521428571 73 0.875 140
1

T5T9	1	-9.6731	-35.7158	3	92	0	0	0	0.097826087		0.064285714
9	0.47826087		0.314285714	44	0.152173913		0.1	14	0.793478261		0.521428571
73	1.52173913		140	0							

DATASET: community

Turtle_ID	porc_nylon	porc_styrofoam	porc_flex	porc_hard	porc_other	area	total
T1T13-20	0.030612245	0.147959184	0.357142857	0.086734694	0.37755102	Beach	1
T1T39	0.007874016	0.275590551	0.362204724	0.173228346	0.181102362	Beach	1
T1T78	0.007874016	0.275590551	0.362204724	0.173228346	0.181102362	Beach	1
T2T102	0.016949153	0.06779661	0.271186441	0.033898305	0.610169492	Beach	1
T2T11-20	0.016949153	0.06779661	0.271186441	0.033898305	0.610169492	Beach	1
T2T17-20	0.056603774	0.188679245	0.188679245	0.226415094	0.339622642	Beach	1
T2T5-20	0.05	0.075	0.2625	0.0375	0.575	Beach	1
T2T62	0.05	0.075	0.2625	0.0375	0.575	Beach	1
T2T86	0.05	0.075	0.2625	0.0375	0.575	Beach	1
T2T95	0	0.072727273	0.490909091	0.109090909	0.327272727	Beach	1
T3T101	0.023809524	0.119047619	0.214285714	0.023809524	0.619047619	Beach	1
T3T113	0.011494253	0.16091954	0.356321839	0.137931034	0.333333333	Beach	1
T3T124	0.018181818	0.163636364	0.327272727	0.109090909	0.381818182	Beach	1
T3T47	0.043478261	0.173913043	0.217391304	0	0.565217391	Beach	1
T3T82	0.011494253	0.16091954	0.356321839	0.137931034	0.333333333	Beach	1
T4T160	0	0.210526316	0.368421053	0.105263158	0.315789474	Beach	1
T4T184	0	0.064285714	0.314285714	0.1	0.521428571	Beach	1
T4T245	0.023809524	0.119047619	0.214285714	0.023809524	0.619047619	Beach	1
T4T284	0	0.064285714	0.314285714	0.1	0.521428571	Beach	1
T4T58	0	0.064285714	0.314285714	0.1	0.521428571	Beach	1
T4T99	0	0.064285714	0.314285714	0.1	0.521428571	Beach	1
T5T115	0	0.064285714	0.314285714	0.1	0.521428571	Beach	1
T5T120	0	0.064285714	0.314285714	0.1	0.521428571	Beach	1
T5T80	0	0.064285714	0.314285714	0.1	0.521428571	Beach	1
T5T9	0	0.064285714	0.314285714	0.1	0.521428571	Beach	1
T1T13-20	0	0	1	0	0	Turtle	1
T1T39	1	0	0	0	0	Turtle	1
T1T78	1	0	0	0	0	Turtle	1
T2T102	0	0	1	0	0	Turtle	1
T2T11-20	1	0	0	0	0	Turtle	1

T2T17-20	0	0	1	0	0	Turtle	1
T2T5-20	0	0	1	0	0	Turtle	1
T2T62	1	0	0	0	0	Turtle	1
T2T86	0	0	1	0	0	Turtle	1
T2T95	1	0	0	0	0	Turtle	1
T3T101	0	0	1	0	0	Turtle	1
T3T113	0	0	1	0	0	Turtle	1
T3T124	0	0	1	0	0	Turtle	1
T3T47	0	0	1	0	0	Turtle	1
T3T82	1	0	0	0	0	Turtle	1
T4T160	0	0	0	0	1	Turtle	1
T4T184	0	0	1	0	0	Turtle	1
T4T245	0	0	1	0	0	Turtle	1
T4T284	1	0	0	0	0	Turtle	1
T4T58	0.3333	0	0.66666	0	0	Turtle	0.99996
T4T99	0	0	1	0	0	Turtle	1
T5T115	1	0	0	0	0	Turtle	1
T5T120	1	0	0	0	0	Turtle	1
T5T80	0	0	1	0	0	Turtle	1
T5T9	0	0	1	0	0	Turtle	1

DATASET: dataset_novo_semNA

Beach	Species	CCL	Plastic_ingestion	N_Items_ingested	Weight_ingested	EPRPE	EPRPEclassification	litter_leakage	State
Ubu	Chelonia_mydas	37.0	1	1	0.01	899	verylow	verylow	ES
PraiadosCastelhianos	Chelonia_mydas	32.8	1	4	0.04	5559	verylow	low	ES
PraiadosCastelhianos	Chelonia_mydas	37.4	1	2	0.01	5559	verylow	low	ES
Itaipava	Chelonia_mydas	35.9	0	0	0	52	verylow	verylow	ES
PraiadoPortinho	Chelonia_mydas	35.8	1	1	0	899	verylow	verylow	ES
PraiaNova	Chelonia_mydas	44.2	1	17	0.12	174	verylow	verylow	ES
Ubu	Chelonia_mydas	32.3	1	15	0.18	899	verylow	verylow	ES
Ubu	Chelonia_mydas	31.5	1	26	0.62	899	verylow	verylow	ES
Ubu	Chelonia_mydas	28.3	1	2	0	899	verylow	verylow	ES
PraiadoCoqueiral	Chelonia_mydas	35.0	1	1	0.01	468	verylow	verylow	ES
EnseadadasGarcas	Chelonia_mydas	35.5	1	5	0.01	100	verylow	verylow	ES
SantaCruz	Chelonia_mydas	45.1	0	0	0	1022	verylow	verylow	ES
SantaCruz	Chelonia_mydas	32.0	0	0	0	1022	verylow	verylow	ES
Itaunas	Chelonia_mydas	34.9	1	6	0.22	7	verylow	verylow	ES
Itaunas	Chelonia_mydas	36.7	0	0	0	7	verylow	verylow	ES
Itaunas	Chelonia_mydas	36.0	1	49	3.03	7	verylow	verylow	ES
Guriri	Chelonia_mydas	35.0	1	53	5.66	83	verylow	verylow	ES
Itaunas	Chelonia_mydas	34.9	0	0	0	7	verylow	verylow	ES
Itaunas	Chelonia_mydas	41.1	1	94	6.77	7	verylow	verylow	ES
Guriri	Chelonia_mydas	38.3	1	25	0.29	83	verylow	verylow	ES
PraiadoRibeiro	Chelonia_mydas	39.2	1	2	0.03	5433	verylow	low	ES
PraiadaCosta	Chelonia_mydas	31.0	1	3	0	5433	verylow	low	ES
IlhadoBoi	Chelonia_mydas	35.7	1	34	1.05	10839	low	low	ES
Itaparica	Chelonia_mydas	31.5	1	2	0.01	5433	verylow	low	ES
CurvadaJurema	Chelonia_mydas	50.5	0	0	0	10839	low	low	ES
CurvadaJurema	Chelonia_mydas	30.2	1	11	0.03	10839	low	low	ES

Camburi	Chelonia_mydas	32.9	1	369	30.75	10839	low	low	ES
CurvadaJurema	Chelonia_mydas	34.7	1	1	0.02	10839	low	low	ES
CurvadaJurema	Chelonia_mydas	40.1	1	1	0	10839	low	low	ES
Itapua	Chelonia_mydas	36.2	1	4	0.09	5433	verylow	low	ES
CurvadaJurema	Chelonia_mydas	33.5	0	0	0	10839	low	low	ES
IlhadoBoi	Chelonia_mydas	38.4	1	2	0.06	10839	low	low	ES
IlhadoBoi	Chelonia_mydas	33.3	1	1	0.01	10839	low	low	ES
IlhadoBoi	Chelonia_mydas	35.3	1	1	0	10839	low	low	ES
IlhadoBoi	Chelonia_mydas	37.3	1	5	0.01	10839	low	low	ES
IlhadoBoi	Chelonia_mydas	42.4	1	10	0.03	10839	low	low	ES
IlhadoBoi	Chelonia_mydas	39.9	1	219	21.01	10839	low	low	ES
BarradoJucu	Chelonia_mydas	33.2	1	2	0	4208	verylow	low	ES
CurvadaJurema	Chelonia_mydas	34.0	1	5	0	10839	low	low	ES
CurvadaJurema	Chelonia_mydas	36.5	0	0	0	10839	low	low	ES
CurvadaJurema	Chelonia_mydas	31.4	1	1	0.02	10839	low	low	ES
CurvadaJurema	Chelonia_mydas	36.2	0	0	0	10839	low	low	ES
CurvadaJurema	Chelonia_mydas	32.0	0	0	0	10839	low	low	ES
CurvadaJurema	Chelonia_mydas	31.7	0	0	0	10839	low	low	ES
PraiadoCanto	Chelonia_mydas	31.7	0	0	0	10839	low	low	ES
PraiadoCanto	Chelonia_mydas	33.0	0	0	0	10839	low	low	ES
CurvadaJurema	Chelonia_mydas	35.1	1	3	0.05	10839	low	low	ES
CurvadaJurema	Chelonia_mydas	58.9	0	0	0	10839	low	low	ES
IlhadoFrade	Chelonia_mydas	42.6	1	2	0.03	10839	low	low	ES
IlhadoFrade	Chelonia_mydas	41.0	0	0	0	10839	low	low	ES
CurvadaJurema	Chelonia_mydas	40.6	1	1	0.04	10839	low	low	ES
CurvadaJurema	Chelonia_mydas	33.2	1	88	2.3	10839	low	low	ES
PraiadoCanto	Chelonia_mydas	37.5	0	0	0	10839	low	low	ES

CurvadaJurema	Chelonia_mydas	35.5	0	0	0	10839	low	low	ES
Camburi	Chelonia_mydas	39.1	0	0	0	10839	low	low	ES
CurvadaJurema	Chelonia_mydas	39.5	0	0	0	10839	low	low	ES
Camburi	Chelonia_mydas	41.3	0	0	0	10839	low	low	ES
CurvadaJurema	Chelonia_mydas	33.5	0	0	0	10839	low	low	ES
Camburi	Chelonia_mydas	42.1	1	4	0.04	10839	low	low	ES
Camburi	Chelonia_mydas	37.0	1	5	0.09	10839	low	low	ES
CurvadaJurema	Chelonia_mydas	36.0	0	0	0	10839	low	low	ES
CurvadaJurema	Chelonia_mydas	32.9	0	0	0	10839	low	low	ES
Iateclube	Chelonia_mydas	35.0	0	0	0	10839	low	low	ES
CurvadaJurema	Chelonia_mydas	36.5	0	0	0	10839	low	low	ES
Jacara pe	Chelonia_mydas	40.0	0	0	0	10839	low	low	ES
EnseadadasGarcas	Chelonia_mydas	32.8	0	0	0	100	verylow	verylow	ES
Iateclube	Chelonia_mydas	31.6	0	0	0	10839	low	low	ES
PraiaGrande	Chelonia_mydas	30.9	0	0	0	689	verylow	verylow	ES
PortodaLama	Chelonia_mydas	31.0	1	2	0.03	100	verylow	verylow	ES
PraiaFormosa	Chelonia_mydas	41.1	1	2	0	100	verylow	verylow	ES
Camburi	Chelonia_mydas	38.2	0	0	0	10839	low	low	ES
Camburi	Chelonia_mydas	60.0	1	20	0.45	10839	low	low	ES
Manguinhos	Chelonia_mydas	45.5	1	1	0	10839	low	low	ES
PraiaGrande	Chelonia_mydas	35.0	1	5	0.02	689	verylow	verylow	ES
PraiaGrande	Chelonia_mydas	37.0	0	0	0	689	verylow	verylow	ES
PraiaGrande	Chelonia_mydas	33.3	1	8	0.11	689	verylow	verylow	ES
EnseadadasGarcas	Chelonia_mydas	31.5	1	1	0	100	verylow	verylow	ES
PraiaGrande	Chelonia_mydas	31.5	1	1	0	689	verylow	verylow	ES
PraiadoBarranco	Chelonia_mydas	36.1	1	1	0	100	verylow	verylow	ES
PraiadoRibeiro	Chelonia_mydas	39.8	1	7	0.03	5433	verylow	low	ES

EnseadadasGarcas	Chelonia_mydas	37.1	1	14	0.08	100	verylow	verylow	ES
SantaCruz	Chelonia_mydas	31.3	1	5	0.02	1022	verylow	verylow	ES
Camburi	Chelonia_mydas	60.5	1	41	0.96	10839	low	low	ES
PraiaGrande	Chelonia_mydas	35.0	1	87	2.89	689	verylow	verylow	ES
PortodaLama	Chelonia_mydas	35.9	1	2	0.01	100	verylow	verylow	ES
PraiaGrande	Chelonia_mydas	29.0	0	0	0	689	verylow	verylow	ES
SantaCruz	Chelonia_mydas	34.5	1	2	0.06	1022	verylow	verylow	ES
PraiadaCosta	Chelonia_mydas	32.0	1	1	0.01	5433	verylow	low	ES
CurvadaJurema	Chelonia_mydas	36.0	1	16	0.07	10839	low	low	ES
CurvadaJurema	Chelonia_mydas	42.7	1	3	0	10839	low	low	ES
PontadaFruta	Chelonia_mydas	33.4	1	2	0	1126	verylow	verylow	ES
Camburi	Chelonia_mydas	46.4	1	49	3.68	10839	low	low	ES
CurvadaJurema	Chelonia_mydas	39.1	0	0	0	10839	low	low	ES
PraiadoCoqueiral	Chelonia_mydas	37.5	1	3	0.03	468	verylow	verylow	ES
PraiadoSaue	Chelonia_mydas	32.1	0	0	0	468	verylow	verylow	ES
PraiadoRioPreto	Chelonia_mydas	34.2	0	0	0	100	verylow	verylow	ES
EnseadadasGarcas	Chelonia_mydas	39.6	1	140	6.66	100	verylow	verylow	ES
CurvadaJurema	Chelonia_mydas	40.1	1	168	4.89	10839	low	low	ES
PraiadoRibeiro	Chelonia_mydas	42.4	1	5	0.04	5433	verylow	low	ES
CurvadaJurema	Chelonia_mydas	38.3	0	0	0	10839	low	low	ES
CurvadaJurema	Chelonia_mydas	38.8	1	1	0	10839	low	low	ES
CurvadaJurema	Chelonia_mydas	32.1	1	3	0.01	10839	low	low	ES
PraiadaJurong	Chelonia_mydas	43.7	0	0	0	468	verylow	verylow	ES
PortodaLama	Chelonia_mydas	34.6	1	40	0.95	100	verylow	verylow	ES
PortodaLama	Chelonia_mydas	35.8	1	7	0.13	100	verylow	verylow	ES
PortodaLama	Chelonia_mydas	38.2	0	0	0	100	verylow	verylow	ES
PortodaLama	Chelonia_mydas	45.2	1	1	0	100	verylow	verylow	ES

Itaparica	Chelonia_mydas	62.8	1	2	0	5433	verylow	low	ES
CurvadaJurema	Chelonia_mydas	39.8	1	1	0.06	10839	low	low	ES
BeiraMar	Chelonia_mydas	47.7	1	9	0.06	10839	low	low	ES
CurvadaJurema	Chelonia_mydas	31.9	1	41	1.46	10839	low	low	ES
BarradoJucu	Chelonia_mydas	37.7	1	90	4.59	4208	verylow	low	ES
PortodaLama	Chelonia_mydas	38.6	1	3	0	100	verylow	verylow	ES
PortodaLama	Chelonia_mydas	31.5	1	13	0.24	100	verylow	verylow	ES
IlhadoBoi	Chelonia_mydas	44.2	1	1	0.01	10839	low	low	ES
PraiaGrande	Chelonia_mydas	31.1	1	2	0	689	verylow	verylow	ES
CurvadaJurema	Chelonia_mydas	42.9	1	1	0.02	10839	low	low	ES
IlhadoBoi	Chelonia_mydas	35.3	1	50	0.75	10839	low	low	ES
PraiadoCapuba	Chelonia_mydas	37.2	0	0	0	446	verylow	verylow	ES
Manguinhos	Chelonia_mydas	37.6	1	1	0	10839	low	low	ES
Camburi	Chelonia_mydas	28.0	1	1	0	10839	low	low	ES
Manguinhos	Chelonia_mydas	40.9	0	0	0	10839	low	low	ES
Manguinhos	Chelonia_mydas	34.3	0	0	0	10839	low	low	ES
PraiadoHavaizinho	Chelonia_mydas	35.7	1	2	0.03	10839	low	low	ES
SantaCruz	Chelonia_mydas	38.8	1	1	0	1022	verylow	verylow	ES
Camburi	Chelonia_mydas	34.6	0	0	0	10839	low	low	ES
Jacara pe	Chelonia_mydas	39.4	0	0	0	10839	low	low	ES
IlhadoFrade	Chelonia_mydas	36.6	1	1	0	10839	low	low	ES
CurvadaJurema	Chelonia_mydas	37.6	1	9	0.04	10839	low	low	ES
PraiadoCoqueiral	Chelonia_mydas	38.4	1	4	0	468	verylow	verylow	ES
Balne rioCarapebus	Chelonia_mydas	31.1	0	0	0	10839	low	low	ES
CurvadaJurema	Chelonia_mydas	46.3	1	1	0	10839	low	low	ES
CurvadaJurema	Chelonia_mydas	41.0	1	1	0	10839	low	low	ES
IlhadoBoi	Chelonia_mydas	36.9	0	0	0	10839	low	low	ES

Jacara pe	Chelonia_mydas	43.3	1	3	0.03	10839	low	low	ES
CurvadaJurema	Chelonia_mydas	44.2	0	0	0	10839	low	low	ES
CurvadaJurema	Chelonia_mydas	34.1	0	0	0	10839	low	low	ES
CurvadaJurema	Chelonia_mydas	39.2	1	2	0.03	10839	low	low	ES
Camburi	Chelonia_mydas	37.8	1	5	0.57	10839	low	low	ES
IlhadaFumaca	Chelonia_mydas	46.9	1	5	0.07	10839	low	low	ES
CurvadaJurema	Chelonia_mydas	39.3	1	1	0	10839	low	low	ES
CurvadaJurema	Chelonia_mydas	44.5	0	0	0	10839	low	low	ES
CurvadaJurema	Chelonia_mydas	36.9	0	0	0	10839	low	low	ES
CurvadaJurema	Chelonia_mydas	36.2	0	0	0	10839	low	low	ES
CurvadaJurema	Chelonia_mydas	42.7	1	1	0.04	10839	low	low	ES
CurvadaJurema	Chelonia_mydas	43.6	0	0	0	10839	low	low	ES
Iateclube	Chelonia_mydas	37.4	0	0	0	10839	low	low	ES
Iateclube	Chelonia_mydas	39.0	1	4	0.05	10839	low	low	ES
Iateclube	Chelonia_mydas	39.1	1	1	0.03	10839	low	low	ES
PraiadoBananal	Chelonia_mydas	51.0	1	2	0.08	5433	verylow	low	ES
CurvadaJurema	Chelonia_mydas	43.8	0	0	0	10839	low	low	ES
Jacara pe	Chelonia_mydas	44.6	0	0	0	10839	low	low	ES
Manguinhos	Chelonia_mydas	37.7	1	1	0.05	10839	low	low	ES
PraiaCoqueiral	Chelonia_mydas	33.4	1	3	0.02	468	verylow	verylow	ES
Prainha	Chelonia_mydas	37.2	1	2	0.01	5443	verylow	verylow	ES
RVS	Chelonia_mydas	42.0	1	5	0.01	1022	verylow	verylow	ES
EnseadadasGarcas	Chelonia_mydas	37.7	1	2	0.01	100	verylow	verylow	ES
IlhadoPapagaio	Chelonia_mydas	52.7	1	7	0.12	10839	low	low	ES
Iateclube	Chelonia_mydas	34.5	0	0	0	10839	low	low	ES
IlhadoBoi	Chelonia_mydas	34.5	1	31	1.31	10839	low	low	ES
Interlagos	Chelonia_mydas	36.1	1	7	0.22	1126	verylow	verylow	ES

EnseadadasGarcas	Chelonia_mydas	34.7	1	24	0.36	100	verylow	verylow	ES
BarradoRiacho	Chelonia_mydas	32.1	1	7	0.16	468	verylow	verylow	ES
BarradoRiacho	Chelonia_mydas	35.6	1	7	0.04	468	verylow	verylow	ES
PraiadoCoqueiral	Chelonia_mydas	35.1	1	4	0.04	468	verylow	verylow	ES
Camburi	Chelonia_mydas	38.0	1	5	0.05	10839	low	low	ES
Prainha	Chelonia_mydas	38.6	1	2	0.01	5443	verylow	verylow	ES
PraiadoCoqueiral	Chelonia_mydas	34.9	1	1	0.02	468	verylow	verylow	ES
NA	Chelonia_mydas	38.4	1	24	0.49	468	verylow	verylow	ES
CurvadaJurema	Chelonia_mydas	34.2	1	2	0	10839	low	low	ES
IlhadoPapagaio	Chelonia_mydas	37.6	1	15	0.45	10839	low	low	ES
CurvadaJurema	Chelonia_mydas	31.5	1	4	0	10839	low	low	ES
CurvadaJurema	Chelonia_mydas	38.2	0	0	0	10839	low	low	ES
PraiaGrande	Chelonia_mydas	54.1	1	4	0.04	689	verylow	verylow	ES
PraiadoCoqueiral	Chelonia_mydas	26.1	1	4	0.03	468	verylow	verylow	ES
PortodaLama	Chelonia_mydas	38.3	1	4	0.03	100	verylow	verylow	ES
CurvadaJurema	Chelonia_mydas	37.0	0	0	0	10839	low	low	ES
CurvadaJurema	Chelonia_mydas	41.3	1	15	0.27	10839	low	low	ES
CostaBela	Chelonia_mydas	37.1	1	1	0.01	446	verylow	verylow	ES
PraiadoCoqueiral	Chelonia_mydas	40.3	1	1	0	468	verylow	verylow	ES
PraiaGrande	Chelonia_mydas	38.1	1	3	0	689	verylow	verylow	ES
Itagu	Chelonia_mydas	37.0	1	20	0.68	8484	low	low	SP
Almada	Chelonia_mydas	40.5	1	4	0.17	8484	low	low	SP
Ten rio	Chelonia_mydas	43.0	1	269	19.34	8484	low	low	SP
Maicabuba	Chelonia_mydas	37.5	1	1	0	8484	low	low	SP
Juquery-SaoSebastiao	Chelonia_mydas	35.5	1	10	0.3	8484	low	low	SP
Itagu	Chelonia_mydas	37.0	1	9	0.37	8484	low	low	SP
Itagu	Chelonia_mydas	55.0	1	7	0.21	8484	low	low	SP

Laz ro	Chelonia_mydas	32.0	1	5	0.02	8484	low	low	SP
Itagu	Chelonia_mydas	32.5	1	1	0.02	8484	low	low	SP
Ubatuba	Chelonia_mydas	32.2	1	4	0.04	8484	low	low	SP
Cedro	Chelonia_mydas	39.0	1	32	0.82	8484	low	low	SP
Hay	Chelonia_mydas	37.5	1	3	0.01	8484	low	low	SP
Itagu	Chelonia_mydas	38.0	1	49	2.01	8484	low	low	SP
Ubatuba	Chelonia_mydas	32.5	1	4	0.06	8484	low	low	SP
Itagu	Chelonia_mydas	44.0	1	9	0.08	8484	low	low	SP
Boqueirao	Chelonia_mydas	49.8	0	0	0	73	verylow	verylow	AL
Bitingui	Chelonia_mydas	37.2	0	0	0	73	verylow	verylow	AL
Japaratinga	Chelonia_mydas	40.3	0	0	0	67	verylow	verylow	AL
Boqueirao	Chelonia_mydas	62.6	0	0	0	73	verylow	verylow	AL
Japaratinga	Chelonia_mydas	39.4	0	0	0	67	verylow	verylow	AL
Maragogi	Chelonia_mydas	49.1	0	0	0	1139	verylow	verylow	AL
Boqueirao	Chelonia_mydas	45.5	1	1	0	73	verylow	verylow	AL
PontadoMangue	Chelonia_mydas	38.1	0	0	0	1139	verylow	verylow	AL
Burgalhau	Chelonia_mydas	40.1	1	1	0	1139	verylow	verylow	AL
Maragogi	Chelonia_mydas	53.5	1	8	0.19	1139	verylow	verylow	AL
SaoBento	Chelonia_mydas	36.3	0	0	0	1139	verylow	verylow	AL
Maragogi	Chelonia_mydas	43.5	1	1	0.38	1139	verylow	verylow	AL
BarraGrande	Chelonia_mydas	64.1	0	0	0	1139	verylow	verylow	AL
Japaratinga	Chelonia_mydas	41.5	0	0	0	67	verylow	verylow	AL
PraiadeBitingui	Chelonia_mydas	42.8	0	0	0	73	verylow	verylow	AL
Boqueirao	Chelonia_mydas	55.1	0	0	0	73	verylow	verylow	AL
Boqueirao	Chelonia_mydas	61.3	1	1	0	73	verylow	verylow	AL
SaoBento	Chelonia_mydas	45.3	1	1	0	1139	verylow	verylow	AL
BarraGrande	Chelonia_mydas	58.9	0	0	0	1139	verylow	verylow	AL

PortodaRua	Chelonia_mydas	61.3	1	1	0	137	verylow	verylow	AL
Lages	Chelonia_mydas	46.0	0	0	0	46	verylow	verylow	AL
Patacho	Chelonia_mydas	53.0	0	0	0	46	verylow	verylow	AL
Lages	Chelonia_mydas	52	0	0	0	46	verylow	verylow	AL
Lages	Chelonia_mydas	53.2	0	0	0	46	verylow	verylow	AL
Tatuamunha	Chelonia_mydas	33.7	0	0	0	137	verylow	verylow	AL
PortodaRua	Chelonia_mydas	56.1	0	0	0	137	verylow	verylow	AL
PortodaRua	Chelonia_mydas	46.3	0	0	0	137	verylow	verylow	AL
Patacho	Chelonia_mydas	42.6	0	0	0	46	verylow	verylow	AL
Lages	Chelonia_mydas	61.5	0	0	0	46	verylow	verylow	AL
PortodaRua	Chelonia_mydas	49.1	0	0	0	137	verylow	verylow	AL
Marcineiro	Chelonia_mydas	41.5	0	0	0	854	verylow	verylow	AL
Tatuamunha	Chelonia_mydas	53.3	0	0	0	137	verylow	verylow	AL
Toque	Chelonia_mydas	42.8	0	0	0	137	verylow	verylow	AL
Lages	Chelonia_mydas	59.7	0	0	0	46	verylow	verylow	AL
Toque	Chelonia_mydas	57.3	0	0	0	137	verylow	verylow	AL
Tatuamunha	Chelonia_mydas	57.1	1	2	0	137	verylow	verylow	AL
Tatuamunha	Chelonia_mydas	43.2	0	0	0	137	verylow	verylow	AL
SaoMigueldosMilagres	Chelonia_mydas	46.5	0	0	0	137	verylow	verylow	AL
Paripueira	Chelonia_mydas	38.0	1	1	0	697	verylow	verylow	AL
SonhoVerde	Chelonia_mydas	46.4	0	0	0	697	verylow	verylow	AL
Mar Mansa	Chelonia_mydas	50.7	0	0	0	190	verylow	verylow	AL
Paripueira	Chelonia_mydas	43.3	0	0	0	697	verylow	verylow	AL
IlhadaCroa	Chelonia_mydas	46.0	1	1	0	190	verylow	verylow	AL
PraiadaIlhadaCroa	Chelonia_mydas	39	0	0	0	190	verylow	verylow	AL
Paripueira	Chelonia_mydas	54.0	0	0	0	697	verylow	verylow	AL
BarradeSantoAnt nio	Chelonia_mydas	33.0	1	1	0	190	verylow	verylow	AL

Tabuba	Chelonia_mydas	50.0	1	2	0	190	verylow	verylow	AL
Paripueira	Chelonia_mydas	35.5	0	0	0	697	verylow	verylow	AL
PedraPreta	Chelonia_mydas	36.5	0	0	0	190	verylow	verylow	AL
SonhoVerde	Chelonia_mydas	55.5	1	2	0.1	190	verylow	verylow	AL
Paripueira	Chelonia_mydas	49.5	0	0	0	697	verylow	verylow	AL
BarradeSantoAnt nio	Chelonia_mydas	63.2	0	0	0	190	verylow	verylow	AL
Mar Mansa	Chelonia_mydas	53	1	1	0.08	190	verylow	verylow	AL
Ipioca	Chelonia_mydas	40.3	0	0	0	697	verylow	verylow	AL
Jacarecica	Chelonia_mydas	41.9	0	0	0	10321	low	low	AL
Guaxuma	Chelonia_mydas	62.5	1	1	0	25500	low	medium	AL
Jacarecica	Chelonia_mydas	47.1	0	0	0	10321	low	low	AL
Jacarecica	Chelonia_mydas	62.4	1	1	0.02	10321	low	low	AL
RiachoDoce	Chelonia_mydas	54.3	0	0	0	25500	low	medium	AL
CruzdasAlmas	Chelonia_mydas	46.4	0	0	0	25500	low	medium	AL
Ipioca	Chelonia_mydas	38.4	0	0	0	697	verylow	verylow	AL
CruzdasAlmas	Chelonia_mydas	57.3	0	0	0	25500	low	medium	AL
MirantedaSereia	Chelonia_mydas	59.5	1	2	0	697	verylow	verylow	AL
Pescaria	Chelonia_mydas	46.0	0	0	0	697	verylow	verylow	AL
Pescaria	Chelonia_mydas	50.3	0	0	0	697	verylow	verylow	AL
Jacarecica	Chelonia_mydas	35.7	0	0	0	10321	low	low	AL
Jacarecica	Chelonia_mydas	58.3	0	0	0	10321	low	low	AL
Jacarecica	Chelonia_mydas	43.6	0	0	0	10321	low	low	AL
Jacarecica	Chelonia_mydas	63.7	0	0	0	10321	low	low	AL
Pescaria	Chelonia_mydas	37.4	0	0	0	697	verylow	verylow	AL
Jacarecica	Chelonia_mydas	53.7	0	0	0	10321	low	low	AL
Jacarecica	Chelonia_mydas	63.5	0	0	0	10321	low	low	AL
Jacarecica	Chelonia_mydas	41.4	0	0	0	10321	low	low	AL

Jacarecica	Chelonia_mydas	43.5	0	0	0	10321	low	low	AL
Ipioca	Chelonia_mydas	45.6	0	0	0	697	verylow	verylow	AL
Jacarecica	Chelonia_mydas	60.1	0	0	0	10321	low	low	AL
Ipioca	Chelonia_mydas	53.4	1	1	0	697	verylow	verylow	AL
MirantedaSereia	Chelonia_mydas	51.3	0	0	0	697	verylow	verylow	AL
Ipioca	Chelonia_mydas	49.7	0	0	0	697	verylow	verylow	AL
Jacarecica	Chelonia_mydas	54.6	0	0	0	10321	low	low	AL
MirantedaSereia	Chelonia_mydas	44.3	0	0	0	697	verylow	verylow	AL
CruzdasAlmas	Chelonia_mydas	38.1	0	0	0	25500	low	medium	AL
CruzdasAlmas	Chelonia_mydas	53.4	0	0	0	25500	low	medium	AL
Pescaria	Chelonia_mydas	48.7	0	0	0	697	verylow	verylow	AL
Pescaria	Chelonia_mydas	44.3	0	0	0	697	verylow	verylow	AL
RiachoDoce	Chelonia_mydas	58.5	0	0	0	25500	low	medium	AL
Ipioca	Chelonia_mydas	56.7	1	1	0	697	verylow	verylow	AL
Ipioca	Chelonia_mydas	64.3	1	1	0	697	verylow	verylow	AL
RiachoDoce	Chelonia_mydas	58.7	1	5	0.23	25500	low	medium	AL
Ipioca	Chelonia_mydas	45.3	0	0	0	697	verylow	verylow	AL
RiachoDoce	Chelonia_mydas	55.4	0	0	0	25500	low	medium	AL
CruzdasAlmas	Chelonia_mydas	64.5	1	5	0.23	25500	low	medium	AL
GarcaTorta	Chelonia_mydas	53.0	1	1	0.19	25500	low	medium	AL
Ipioca	Chelonia_mydas	65.0	0	0	0	697	verylow	verylow	AL
Ipioca	Chelonia_mydas	52.2	0	0	0	697	verylow	verylow	AL
RiachoDoce	Chelonia_mydas	45.5	0	0	0	25500	low	medium	AL
RiachoDoce	Chelonia_mydas	50.8	0	0	0	25500	low	medium	AL
Ipioca	Chelonia_mydas	53.5	0	0	0	697	verylow	verylow	AL
RiachoDoce	Chelonia_mydas	43.7	1	3	0	25500	low	medium	AL
Ipioca	Chelonia_mydas	36.5	0	0	0	697	verylow	verylow	AL

Ipioca	Chelonia_mydas	51.3	0	0	0	697	verylow	verylow	AL
CruzdasAlmas	Chelonia_mydas	34.9	1	13	0	25500	low	medium	AL
PontaVerde	Chelonia_mydas	61.3	0	0	0	25500	low	medium	AL
PontaVerde	Chelonia_mydas	59.2	0	0	0	25500	low	medium	AL
Pajucara	Chelonia_mydas	59	1	2	0	25500	low	medium	AL
Pajucara	Chelonia_mydas	62.3	1	1	0	25500	low	medium	AL
Jatiuca	Chelonia_mydas	51.7	0	0	0	25500	low	medium	AL
PontaldaBarra	Chelonia_mydas	46.0	1	3	0	21417	low	medium	AL
PontaVerde	Chelonia_mydas	47.1	0	0	0	25500	low	medium	AL
Jatiuca	Chelonia_mydas	45.1	0	0	0	25500	low	medium	AL
Jatiuca	Chelonia_mydas	47.2	0	0	0	25500	low	medium	AL
Jatiuca	Chelonia_mydas	42.3	1	2	0	25500	low	medium	AL
Pajucara	Chelonia_mydas	59	0	0	0	25500	low	medium	AL
Jatiuca	Chelonia_mydas	62.0	0	0	0	25500	low	medium	AL
Jatiuca	Chelonia_mydas	40.0	0	0	0	25500	low	medium	AL
Pajucara	Chelonia_mydas	44.6	1	3	0	25500	low	medium	AL
Pajucara	Chelonia_mydas	40.3	0	0	0	25500	low	medium	AL
Jatiuca	Chelonia_mydas	55.5	0	0	0	25500	low	medium	AL
PontaVerde	Chelonia_mydas	56.3	1	1	0	25500	low	medium	AL
Pajucara	Chelonia_mydas	62.3	0	0	0	25500	low	medium	AL
Jatiuca	Chelonia_mydas	36.1	0	0	0	25500	low	medium	AL
Pajucara	Chelonia_mydas	48.5	1	1	0	25500	low	medium	AL
Jatiuca	Chelonia_mydas	46.5	0	0	0	25500	low	medium	AL
BarradeSaoMiguel	Chelonia_mydas	42.3	1	3	0	441	verylow	verylow	AL
PraiaBonita	Chelonia_mydas	37	0	0	0	441	verylow	verylow	AL
Prainha	Chelonia_mydas	63.0	0	0	0	4980	verylow	verylow	AL
Gunga	Chelonia_mydas	52.0	1	9	0	15	verylow	verylow	AL

Gunga	Chelonia_mydas	59	0	0	0	15	verylow	verylow	AL
Gunga	Chelonia_mydas	41.2	1	15	0	15	verylow	verylow	AL
IlhadoPrinc pe	Chelonia_mydas	54.4	1	1	0	10839	low	low	ES
PortodeVit ria	Chelonia_mydas	32.5	1	8	0.04	10839	low	low	ES
IlhadoBoi	Chelonia_mydas	35.2	1	142	3.85	10839	low	low	ES
IlhadoBoi	Chelonia_mydas	32.4	1	63	0.59	10839	low	low	ES

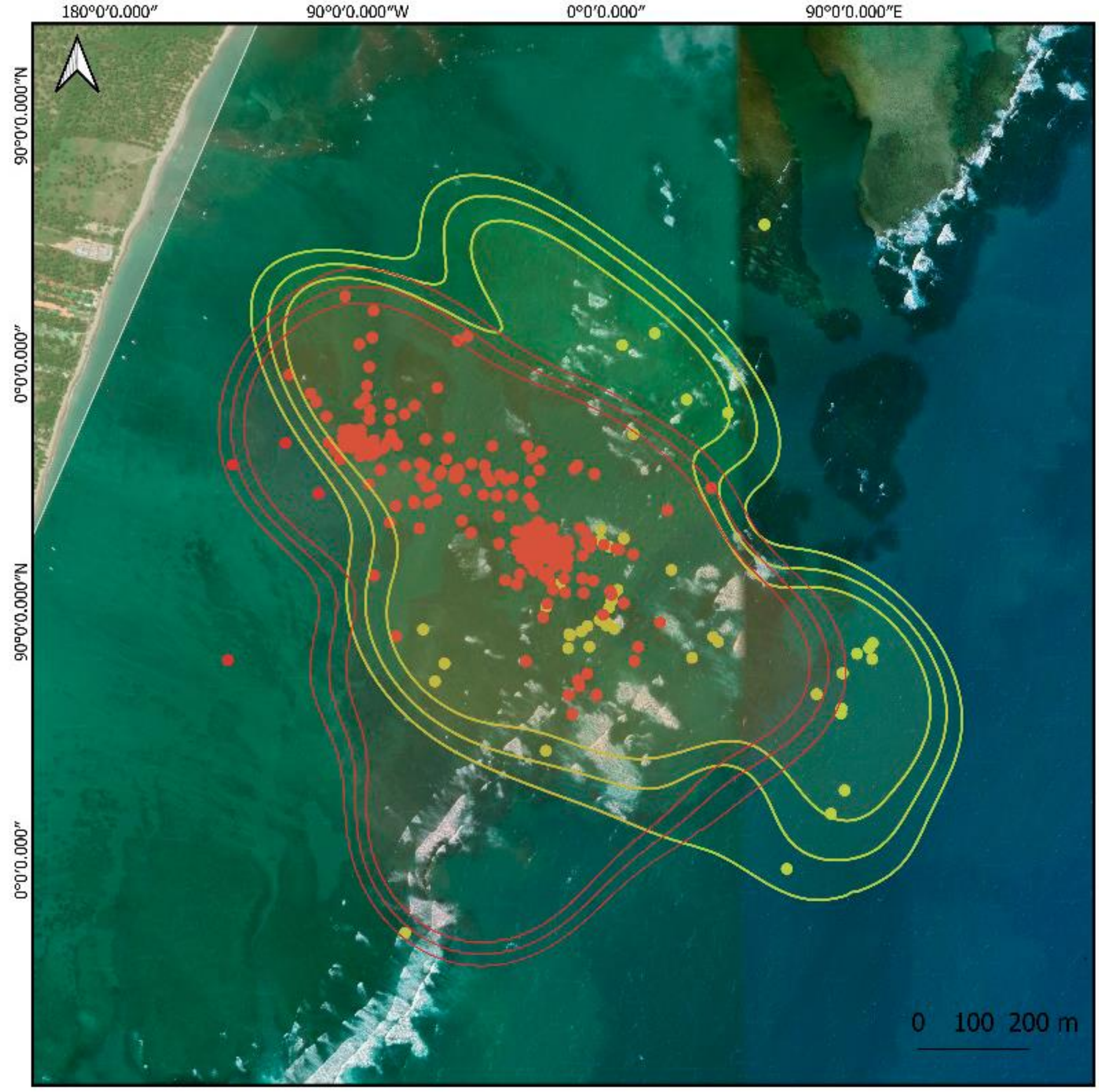

Figure S1 – Home ranges of two green turtles (*Chelonia mydas*) tracked via satellite telemetry (model: Lotek FastGPS ARGOS series F6G-276B) in the study area (local approach – Porto de Pedras, Alagoas, Brazil). Yellow: green turtle captured in March 2022, curved carapace length 35.5 cm, tracked for 110 days. Red: green turtle captured in September 2023, curved carapace length 47.2 cm, tracked for 29 days.

Local approach – No difference of plastic on the beaches overtime

To verify if the plastic density varied along the time, we built generalized linear mixed models in which the plastic density was the response variable, and the year was the fixed predictor variable. We used the Transect ID nested in the Month as the random factor. The model was built using poisson distribution, to account for the heavily skewed.

Table S1. Plastic density on the beaches over time.

Year	Mean	SD	Min	Max
2017	1.7	1.21	0.12	5.65
2018	1.54	1.25	0.25	6.25
2019	1.02	0.82	0.05	4.2
2020	1.76	1.73	0.05	12.73
2021	1.11	0.98	0.09	4.27
2022	0.79	0.43	0.05	2.03
2023	2.15	1.7	0.18	8.91

The mean plastic density in the beaches during the sampling period was 1.44 Ñ1.30 plastic items/mĮ. The mean density values per year ranged from 0.79 Ñ0.43, to 2.15 Ñ1.7(Table S1). The sampling year did not affect plastic density in the beaches (GĮ = 0,0008, d.f. = 1, p = 0.9776), and plastic environmental availability remained constant along the sampled period. We assumed this condition to be true for all the sampled areas.

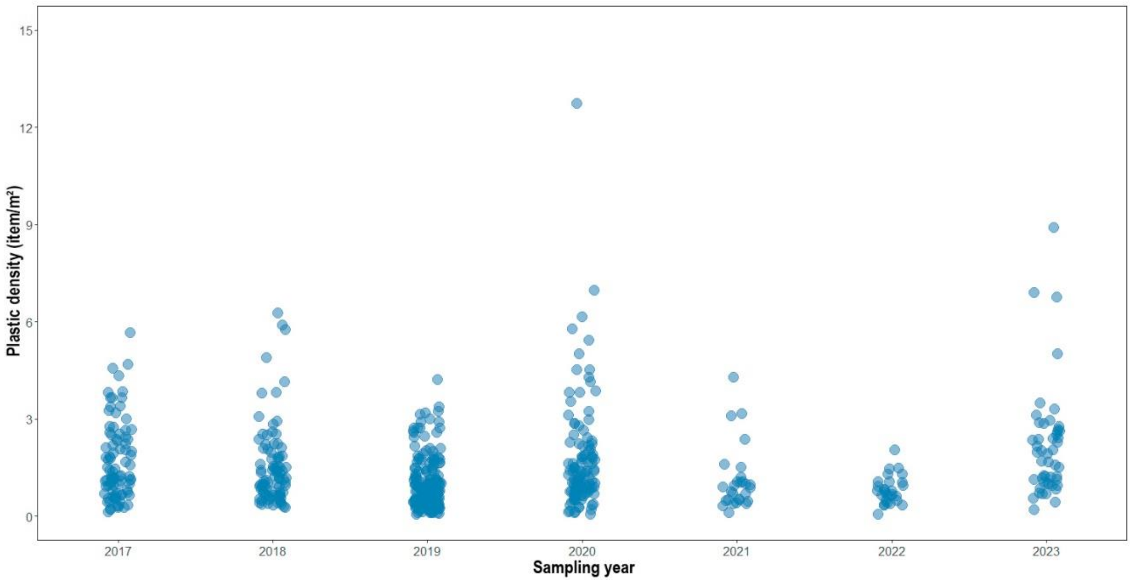

Figure S2 ï Plastic density (items/mĮ) per transect per year at the beaches.

Detailed statistical results: Relationship between plastic availability and plastic ingestion

Table S2. Results of GLM model showing that CCL and probability of plastic ingestion are negatively correlated.

Source of variation	Estimate	Std. Error	t value	*p* value
Intercept	2.3299	0.5570	4.183	<0.001
CCL	-0.0509	0.0182	0.012	<0.001

Table S3. Results of GLM model showing that CCL and the number of plastic items ingested by green turtles are negatively correlated.

Source of variation	Estimate	Std. Error	t value	*p* value
Intercept	4.7965	1.1123	4.312	<0.001
CCL	-0.0652	0.0286	-2.282	0.0231

Table S4. Results of GLM model showing the correlation between turtles CCL and the number of plastic items ingested by green turtles.

Source of variation	Estimate	Std. Error	t value	*p* value
Intercept	2.3748	0.79825	2.975	<0.001
CCL	-0.0318	0.01503	-2.115	0.042

Table S5. Results of GLM model showing the correlation between plastic categories and the number of plastic items ingested by green turtles. Other: glass, rubber metal, cloth, foam and cigarette buts.

Source of variation	Estimates	Std. Error	t value	*p* Value
Intercept	-0.4599	0.3810	-1.207	0.2294
Styrofoam	-28.3896	8.2307	-3.449	<0.001
Flexible plastic	17.3326	4.2524	4.076	<0.001
Other	-6.9784	2.1335	-3.271	<0.001
